# Regenerative strategies for intervertebral disc degeneration

**DOI:** 10.1016/j.jot.2025.06.003

**Published:** 2025-07-04

**Authors:** Raed H. Ogaili, Ahmed Alassal, Nurul Fariha Za'aba, Izzat Zulkiflee, Isma Liza Mohd Isa

**Affiliations:** a CÚRAM Research Ireland Centre for Medical Devices, University of Galway, Galway, H91 W2TY, Ireland; b Department of Anatomy, Faculty of Medicine, Universiti Kebangsaan Malaysia, Cheras, 56000, Kuala Lumpur, Malaysia; c College of Veterinary Medicine, University of Karbala, Karbala, 65001, Iraq; d Riverside School of Medicine (UCR-SOM), University of California, Riverside, 900 University Ave, Riverside, CA, 92521, USA; e Pharmacology and Therapeutics, School of Medicine, University of Galway, Galway, H91 TK33, Ireland

**Keywords:** Intervertebral disc degeneration, regenerative medicine, Mesenchymal stem cells, Biomaterials

## Abstract

Low back pain (LBP) is a global health problem, primarily caused by intervertebral disc (IVD) degeneration. Current treatments focus on symptom relief without addressing the underlying degenerative mechanisms. Regenerative strategies have emerged as promising therapies through the use of functional biomaterials and stem cells capable of modulating key signalling pathways to promote tissue regeneration. However, challenges such as efficient delivery systems, long-term survival of transplanted cells, and hostile disc microenvironment remain. This review focuses on recent advances in regenerative approaches using biomaterials, cells, and therapeutic agents of exosomes, and genes to restore IVD structure and function. We discuss the current understanding of IVD anatomy, physiology and degeneration pathophysiology followed by current treatments. We highlight the rationale for regenerative therapy in halting the degenerative hallmarks tailored to mild, moderate to severe IVD degeneration. Our review emphasizes on the functional biomaterials designed for advanced delivery system, therapeutic intervention and IVD tissue engineering. We discuss the cell-based therapy, highlighting various cell sources, therapeutic effects, clinical trials and its obstacles. We discuss the use of therapeutic agents such as the genes and exosome therapies in IVD regeneration. The clinical translational potential of regenerative therapy is vast and promising, driven by advances in cellular therapies, biomaterials, and cell-free approaches like exosomes, which offer new avenues for regenerating degenerative IVDs. While significant progress has been made in developing safe, effective, and scalable treatments, challenges remain in immune compatibility, manufacturing, and regulatory pathways. Emerging innovations in gene editing, 3D bioprinting, and personalized approaches are poised to accelerate the translation of these therapies into mainstream medicine, with interdisciplinary collaboration and global efforts playing a crucial role in overcoming current bottlenecks and realizing the full potential of regenerative medicine to transform patient care. This article offers a comprehensive framework to guide preclinical research and future clinical translation of effective regenerative therapies, aiming at reducing the global burden of LBP and improving long-term patient outcomes.

## Graphical abstract

**Figure:**
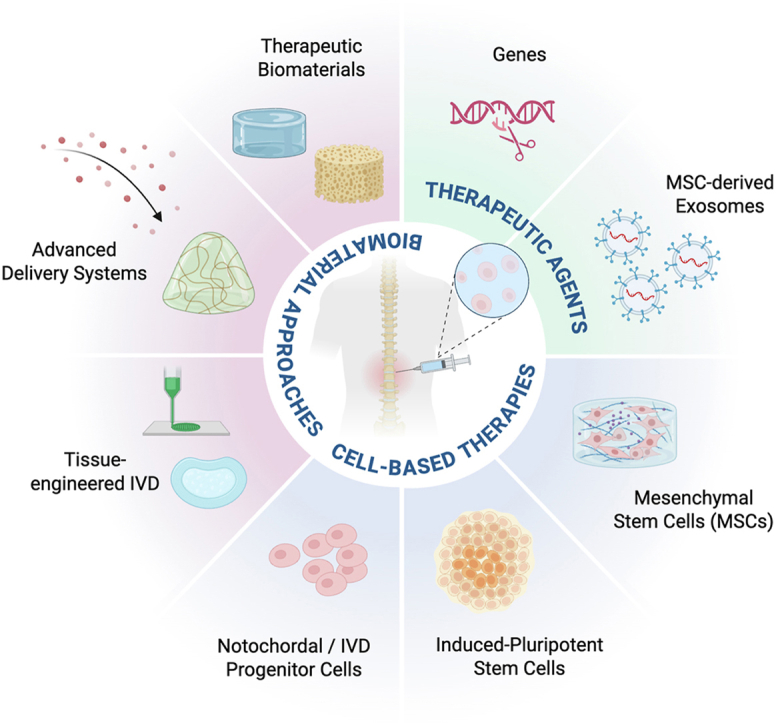

## 1 Introduction

Intervertebral disc (IVD) degeneration is the leading cause of discogenic lower back pain (LBP). LBP causes significant problems for people worldwide. According to the global burden of disease studies, LBP accounts for a significant portion of disability-adjusted life years (DALYs) globally. Degenerative disc disorders associated with LBP impact around 632 million individuals, accounting for almost 40 % of all LBP occurrences [1].

The IVD degeneration represents an inevitable component of the aging process, though its pathological progression can be attributed to a multifactorial aetiology encompassing genetic predisposition, mechanical stress, and environmental determinants. These factors collectively precipitate a reduction in cellular density and perturbation of extracellular matrix (ECM) homeostasis, subsequently inducing inflammatory cascades. The resultant inflammatory milieu facilitates the pathological innervation of nociceptive nerve fibers into the typically aneural IVD, culminating in pain manifestation [2].

This comprehensive review elucidates the anatomical architecture, pathophysiological mechanisms, and current therapeutic interventions for IVD degeneration. Particular emphasis is placed on emerging regenerative approaches, specifically the application of stem cell therapy, genetic modification strategies, and biomaterial scaffolds for tissue regeneration and restoration of disc function.

## 2 Anatomy of the intervertebral disc

**Fig. 1:**
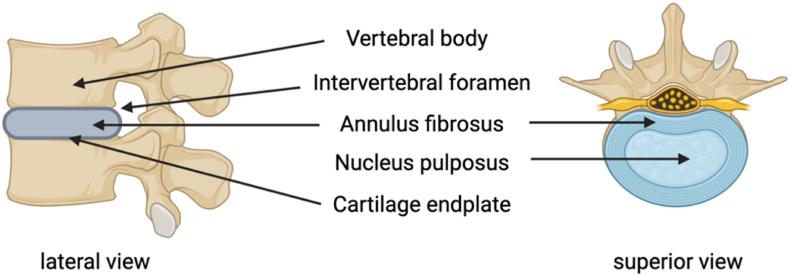
Anatomy of the IVD, emphasizing the relationship between the annulus fibrosus, nucleus pulposus, cartilage endplate, and adjacent vertebrae. Schematic was created by BioRender.

The IVD is classified as a symphysis joint, a fibrocartilage spacer between two adjacent vertebral bodies that facilitates movement and load distribution. Its component distributes pressure evenly for all the vertebral bodies, even if the spine is bent or stretched [3] The IVD is comprised of the nucleus pulposus (NP), annulus fibrosus (AF), and cartilaginous endplates (CEP) [4,5] (Fig. 1).

The nucleus pulposus (NP) is the central core of the IVD. The dry weight of the IVD ranges between 35 and 65 % proteoglycan, 5–20 % fine type II collagen fibrils, and some non-collagenous proteins and elastin. It is composed of 70–90 % water. The NP allows for disc mobility and the ability to absorb mechanical energy during compressive stress. The NP in a normal disc is forward and may touch the surrounding tissues and epidural space during lumbar extension. The radial and axial distribution of elastin fibres in the nucleus pulposus makes them optimal for mending a bent disc. The ability of the NP to withstand compression is associated with the water retention capacity of its proteoglycan content. During ageing, the proteoglycan aggregate content reduces its capacity [6]. The ECM of the NP is made up of collagen II and elastin fibres embedded in a gel containing aggrecan. The NP cells have a sparse shape and are made up of a combination of tiny mesenchymal cells similar to chondrocytes and larger notochordal cells thought to have originated from the notochord. During intervertebral disc formation, the massive, highly vacuolated notochordal cells undergo morphological and functional modifications, resembling smaller, fibrochondrocyte-like cells [7].

The fibrous ring around the NP is known as the annulus fibrosus (AF). It is made up of 15–25 concentric layers of highly structured collagen fibres called lamellae. Each lamella is made up of oblique collagen strands. The IVD can resist circumferential stresses while limiting rotation and bending between neighbouring vertebrae due to the oblique crossing of fibres in adjacent lamellae. Its dry weight is composed of 20 % collagen, 2 % elastin, and 20 % proteoglycan. The thickness of each lamella ranges from 100 to 500 μm, with exterior lamellae being thicker than interior. Each lamella is separated by an interlamellar tissue comprising cells, elastic fibres, and a proteoglycan-rich matrix. The annulus fibrosus (AF) is separated into two regions: the inner AF contains type II collagen fibers, mostly formed by chondrocyte-like cells with a rounded appearance. The outer annulus fibrosus mostly comprises type I collagen fibres generated by elongated, spindle-shaped fibroblast-like cells of mesenchymal origin [8].

The cartilaginous end plate (CEP) is a 0.6 mm layer of hyaline cartilage that connects the vertebral body above to the NP below [9]. The CEP comprises 60 % proteoglycan, collagen type II, and water. The CEP was made up of elongated collagen-producing cells oriented parallel to the IVD and collagen fibres. With age, the CEP becomes avascular, functioning as a physical barrier between the NP and the spine while simultaneously allowing nutrients to reach the IVD via surrounding blood vessels [10]. High horizontal strains may cause segmental separation of the end plate due to a poor connection between the CEP and the underlying vertebral body [9,11,12].

Small arterioles penetrate through the cartilaginous endplate to provide blood to the intervertebral disc during infancy. In adults, the IVD receives blood from two distinct capillary networks. One begins at the vertebral bodies and ends at the bone-cartilage junction, while the other transports blood to the AF's outer surface. The intervertebral disc's avascular structure is supplied with nutrients via diffusion [13]. The NP is a tissue that lacks nerve supply and blood arteries, depending on diffusion to deliver nutrients and oxygen and remove waste from metabolic activities. It is comparable to articular hyaline cartilage in these two ways. The sinuvertebral nerves (SVN) innervate mostly the outer section of the AF and are composed of an autonomic root from the grey ramus and a somatic root from the ventral ramus, while NP lacks nerve innervation [14,15].

## 3 Pathophysiology of the intervertebral disc degeneration

**Fig. 2:**
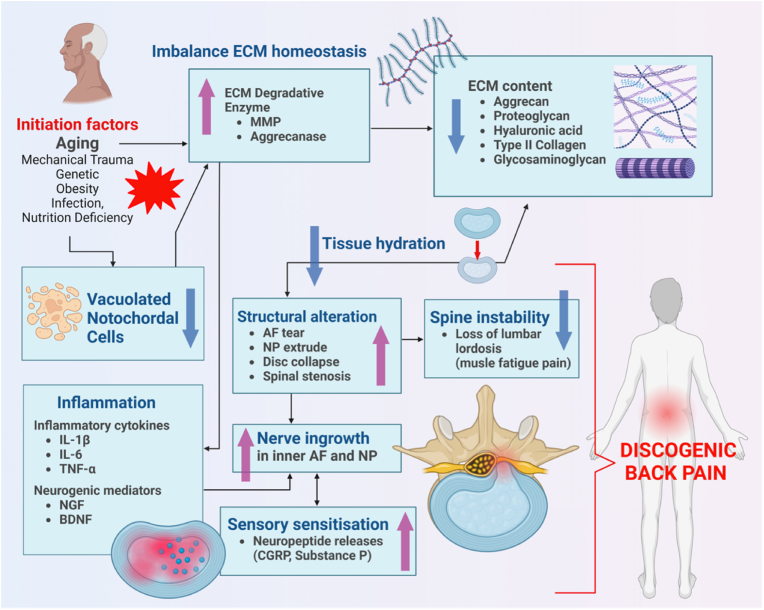
Pathophysiology of IVD degeneration, including the ECM imbalance, inflammation, structural changes, neovascularization, nerve ingrowth, sensory mediated nociception and spine instability. The onset of IVD degeneration is mediated with alteration in cellular phenotype and functions includes loss of notochordal cells, resulting in apoptosis, senescence and an imbalance in ECM metabolism, indicating by an increase of degradative enzymes (e.g., aggrecanases, MMPs) and decreased synthesis of ECM components (e.g., aggrecan, collagen, proteoglycans). ECM dysregulation triggers the release of proinflammatory cytokines (e.g., IL-1β, TNF, IL-6) to mediate inflammation in the IVD. Prolonged inflammation results in ECM breakdown, reduces cell density, and promotes the release of neurogenic mediators like VEGF, NGF, and BDNF by disc and immune cells, leading to neovascularization and sensory nerve ingrowth into aneural IVD. Sensitisation of sensory nerves through pronociceptive signalling (e.g., neuropeptides release), resulting in discogenic pain. Advanced degeneration causes structural alteration, including annular tears and disc collapse often leading to spine mechanical instability. Figure adaptation from Razak et al., 2024.

Intervertebral disc (IVD) degeneration is a progressive and multifactorial process that contributes to lumbar spine dysfunction and instability. Both biological and biomechanical factors play a role in its progression, leading to pain and associated symptoms. The primary mechanisms driving IVD degeneration include inflammation, mechanical instability, and vascular alterations (Fig. 2), which interact with various intrinsic and extrinsic risk factors to accelerate degenerative changes.

### 3.1 Cellular phenotypic change, matrix dysregulation and inflammation

The initial stage of IVD degeneration is characterized by a decline in large vacuolated notochordal cells in the nucleus pulposus (NP). The expression of notochordal markers, such as Brachyury, has been associated with glycosaminoglycan deposition and a reduction in inflammatory markers, including interleukin (IL)-1β, IL-6, and nerve growth factor (NGF) [16]. These inflammatory cytokines drive the production of matrix-degrading enzymes, including matrix metalloproteinases (MMPs) and aggrecanases (ADAMTS-4 and ADAMTS-5) [17]. These enzymes degrade essential extracellular matrix (ECM) components such as hyaluronic acid, type II collagen, glycoproteins, and elastic fibers, contributing to structural deterioration [18].

As the matrix composition shifts, there is a decrease in type II collagen synthesis and an increase in type I collagen production, along with enhanced collagen fibre orientation and matrix-degrading enzyme activity. This results in loss of disc elasticity, reduced hydration, and mechanical instability [19]. Disruptions in extracellular vesicle (EV) signalling further exacerbate the imbalance between ECM synthesis and degradation, contributing to degenerative progression [20]. The loss of proteoglycans in the NP creates an environment conducive to the infiltration of pro-inflammatory cytokines, serum proteins, and neurogenic mediators, perpetuating inflammation [21]. NP and annulus fibrosus (AF) cells, along with macrophages, T cells, and neutrophils, secrete high levels of pro-inflammatory cytokines such as tumour necrosis factor (TNF)-α, interferon-γ (IFN-γ), IL-1β, IL-10, IL-4, IL-6, IL-17, IL-2, IL-8, and chemokines like CCR6 and CCL20. These molecules further promote inflammation-driven disc degeneration.

Interleukin-1 (IL-1) is a major pro-inflammatory cytokine that induces matrix degradation in intervertebral discs (IVDs) by upregulating matrix metalloproteinases (MMPs) and aggrecanases, leading to extracellular matrix (ECM) breakdown and disc degeneration [22]. Tumor necrosis factor-alpha (TNF-α) is crucial in promoting inflammation, inducing disc cell apoptosis, and stimulating catabolic enzyme expression, all of which accelerate IVDD progression [23]. Interleukin-6 (IL-6) modulates the inflammatory environment within the IVD and can further enhance catabolic activities, influencing pain and degeneration severity [24].

Matrix metalloproteinases (MMPs) are enzymes responsible for degrading ECM components in the intervertebral disc. In healthy discs, MMP activity is tightly controlled, but in intervertebral disc degeneration (IVDD), MMPs become overexpressed, leading to the breakdown of key structural proteins like type II collagen and aggrecan, weakening the disc's ability to retain water and bear mechanical loads. Inflammatory cytokines like IL-1β and TNF-α further stimulate MMP production, accelerating degeneration. Key MMPs involved include MMP-1, MMP-3, and MMP-13. Targeting MMPs is a potential therapeutic strategy to slow or prevent disc degeneration [25,26].

### 3.2 Annular tear, neovascularization and nerve in-growth into aneural disc

As degeneration progresses, the loss of joint space results in significant mobility impairment. Structural deterioration of the disc leads to biomechanical failure, with fissures in the annulus fibrosus allowing the nucleus pulposus to herniate. This promotes the ingrowth of sensory nerves and blood vessels into the inner annulus fibrosus and NP, contributing to discogenic back pain [27].

The interplay between inflammation and disc degeneration is crucial in understanding the pain associated with IVD degeneration. Degenerated IVDs exhibit a proinflammatory milieu, evidenced by upregulation of TNF-α, IL-1β, IL-6, IL-8, and pain-related neuropeptides such as substance P. Inflammatory mediators not only promote ECM degradation but also induce pain-related factors such as nitric oxide (NO), cyclooxygenase-2 (COX-2), and NGF. NGF, in particular, plays a key role in enhancing sensory innervation and nociceptive nerve sensitivity, results in nociception [28]. Nociceptive pain associated with IVD degeneration is driven by the ingrowth of sensory nerve fibers into the degenerated disc matrix. The presence of inflammatory, pronociceptive mediators, and mechanical loading sensitize afferent nociceptors, leading to pain signal transduction. These nociceptive signals are relayed via primary afferent neurons to the dorsal horn of the spinal cord, projecting to thalamus towards somatosensory area in the brain for pain processing – explain the contribution of inflammation, innervation and sensory sensitisation to the onset and persistence of discogenic pain. The increase in vascularization in response to inflammation facilitates immune cell infiltration, thereby maintaining the inflammatory cycle. Neovascularization in the NP further exacerbates pain due to the sensitisation of nerve fibers by inflammatory cytokines, particularly through vascular endothelial growth factor (VEGF) [29,30].

### 3.3 Disc dehydration, mechanical instability and osteophyte formation

**Fig. 3:**
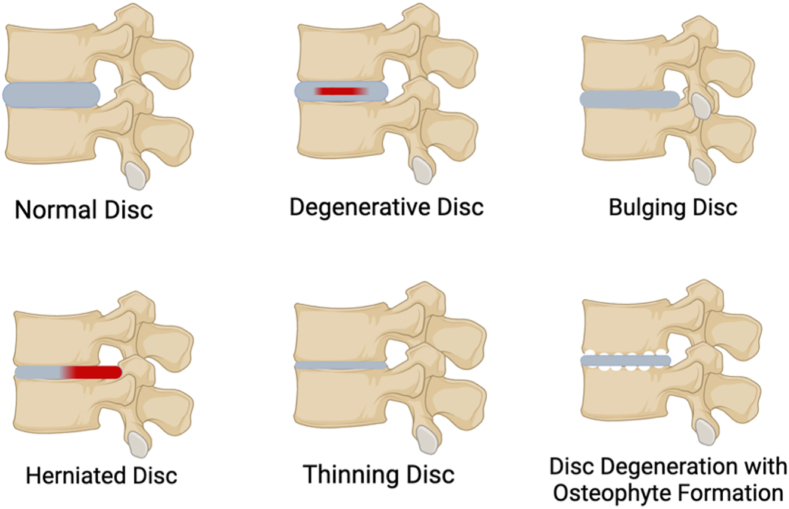
Structural changes in the IVD, highlighting the degenerative feature, bulging, herniated, disc collapse and osteophyte formation. Schematic was created by BioRender.

Advanced stages of IVD degeneration are characterized by dehydration and loss of disc height, leading to mechanical instability and altered spinal biomechanics. Increased stress on adjacent vertebrae promotes osteophyte formation, a process that further impairs spinal function [31]. Annular tears facilitate the extrusion of NP material, worsening nerve compression and leading to heightened pain symptoms. Chondroitin sulfate (CS) and its associated enzymes, xylosyltransferase I (XT-I) and glucuronyl transferase I (GT-I), play a critical role in maintaining glycosaminoglycan (GAG) production in healthy discs. The decline in these enzymes during degeneration is linked to reduced CS levels, which compromises disc hydration and mechanical function [32,33]. These interconnected processes mirror the complexity of intervertebral disc degeneration. Fig. 3 shows the structural changes in the IVD, highlighting the degenerative feature, bulging, herniated, disc collapse and osteophyte formation. Overall, the role of inflammatory mediators, degradation enzymes, pain markers and vascularization reflect their contribution to structural changes, sensory sensitisation and mechanical instability in the intervertebral disc.

In conclusion, Intervertebral disc degeneration is a complex process influenced by inflammatory mediators, ECM degradation, pain-associated markers, and neovascularization. These factors contribute to structural changes, sensory sensitisation, and mechanical instability, ultimately leading to lumbar spine dysfunction. Understanding these intricate mechanisms is essential for developing targeted therapeutic interventions to mitigate the progression of IVD degeneration.

## 4 Current treatments

The treatment of low back pain includes both pharmacological and nonpharmaco-logical therapies. Most common pharmacological drugs to treat pain are non-steroidal anti-inflammatory drugs (NSAIDs) which do so by decreasing the inflammation in the pathways of pain. COX-2 inhibitors are a kind of NSAID that targets COX-2 enzymes to decrease inflammation and pain with less side effects on the gastrointestinal system than nonselective NSAIDs. NSAIDs can be used in concert with other pain relievers, such as paracetamol and moderate opioids (e.g., tramadol), to treat pain via several routes. Additional pharmaceutical treatments for neuropathic pain include anticonvulsants and/or antidepressants such as gabapentin, pregabalin, and duloxetine [10].

Nonpharmacological therapy consists mostly of a rehabilitation program meant to return patients to their normal functional level, including increased range of motion, while preventing further injury. This can be performed by improving core muscle strength, endurance, and coordination. Current multimodal rehabilitation treatments mostly combine exercise treatment with cognitive behavioural training, which is more effective in reducing disability and fear associated with pain than exercise therapy alone. Efficient rehabilitation will reduce dependency on medications [34].

Surgery will be the final step in addressing degenerative disc degeneration. Early decompression surgery is appropriate in the case of cauda equina syndrome. Decompression surgery improves outcomes in individuals with moderate to severe degenerative spinal stenosis, particularly those who have significant leg pain but little instability. Decompression with fusion is suggested when instability is proven prior to surgery or following a decompression procedure. With an increasing life expectancy, more older people are being diagnosed with primary degenerative sagittal imbalance. The optimum surgical strategy will focus on addressing spinal column defects, such as sagittal imbalance, using operations such as spinal osteotomy, spinal instrumentation, and fusion. Corrective surgery for this sagittal anomaly is more common, and the approach has shown promising outcomes in certain groups [34].

## 5 Biomarkers in intervertebral disc

Biomarkers are quantitative markers of a biological degenerative state. They can also suggest the degenerative, normal and therapeutic intervention responses within biology. Dictionary - A molecular marker is an observable trait (phenotype) of a molecule that can be used as an indicator of genomic characteristics; thus, the expression patterns of pro-inflammatory, anti-inflammatory, progenitor, and extracellular matrix molecular markers can allow us to define new therapeutic targets and provide a better understanding of the underlying mechanisms of disc degeneration [35]. It may also provide valuable insight into the development and severity of intervertebral disc degeneration with the analysis of changes in extracellular matrix component using molecular markers [36]. This information could also greatly accelerate the creation of targeted drugs and therapies that would likely aim to alleviate the prognosis of patients with disc degeneration [37].

Table 1 summarises the key markers expressed in the AF and NP. The ECM markers include collagen type II (COL2A1), aggrecan (ACAN), collagen type I (COL1A1), tissue inhibitors of metalloproteinases (TIMPs) and matrix metalloproteinases (MMPs) [38]. Cellular senescence markers (CDKN2A) and inflammatory markers have been widely discovered, including (IL-1, IL6, and TNF-α) [39,40]. NP marker of SOX9 and notochordal cell markers such as CK8, CK18 and brachyury [41]. The progenitor surface markers (Tie2^+^, GD2^+^, Nanog^+^, Oct-4^+^, Sox-2^+^, CD44^+^, Notch1^+^, Delta4^+^, CD117^+^, STRO-1^+^) have been found in the NP. Similarly, the AF has also expressed progenitor surface markers, including the CD24^+^, Stro-1^+^, Nestin^+^, NSE^+^, and CD44^+^, indicating that these cells may play an important role in disc regeneration and repair [42].

**Table 1:** Potential biomarkers of the mature intervertebral disc.

Biomarkers	Description	Refs
Collagen Type II (COL2A1)	A major structural protein in the nucleus pulposus and annulus fibrosus of the intervertebral disc.	[43]
Aggrecan (ACAN):	A large proteoglycan responsible for maintaining hydration and resilience in the disc.	[44,45]
Sox9 (SRY-Box Transcription Factor 9)	A transcription factor associated with chondrogenesis and expressed in cells within the nucleus pulposus.	[45]
Brachyury	A transcription factor associated with notochordal cells, which play a role in the development of the intervertebral disc.	[41]
Collagen Type I (COL1A1)	Predominantly found in the annulus fibrosus, providing tensile strength to the disc.	[43]
Matrix Metalloproteinases (MMPs)	MMP-1, MMP-2, MMP-3, MMP-9, and MMP-13 are enzymes associated with extracellular matrix remodelling and are often upregulated in degenerating discs.	[46]
Tissue Inhibitors of Metalloproteinases (TIMPs)	TIMP-1, TIMP-2, and other TIMPs regulate the activity of MMPs, maintaining a balance in matrix turnover.	[46]
Notochordal Cell Markers (CK8, CK18)	Cytokeratin 8/18: A marker for notochordal cells in the nucleus pulposus.	[47]
Inflammatory Markers (IL-1, IL6, TNF-α)	Interleukin-1 (IL-1), IL-6, Tumour Necrosis Factor-alpha (TNF-α): Pro-inflammatory cytokines associated with disc degeneration and inflammation.	[39]
Cellular Senescence Markers (CDKN2A)	p16INK4a (CDKN2A): A marker associated with cellular senescence, which can occur in aging or degenerating intervertebral discs.	[40]
Hyaluronan Receptor (CD44)	Associated with mesenchymal stem cells in the intervertebral disc.	[48]
Growth Factors (TGF-β)	Transforming Growth Factor-beta (TGF-β): Involved in cell differentiation and matrix synthesis in the intervertebral disc.	[46]
Catabolic Molecules (IL-1A, IL-6)	IL1A - 889 T allele represented a significant risk factor for IVD degeneration.	[49]
	IL-6 Single nucleotide polymorphism is involved in the aetiology of IVD degeneration among young adults (Association of IL-6 genetic variations with discogenic pain)	[50]

## 6 Rationale of regenerative therapy in degenerative discs

As the current clinical treatments are not regenerative in nature, regenerative medicine aims to improve tissue or organ function through replacement, repair, or restoration. Each technique is at different stages of development and has had varying degrees of success in preclinical and/or clinical trials. Regenerative strategies include cell-, biomaterial- and gene-based therapies have emerged as a promising avenue for addressing the fundamental pathophysiological mechanisms of IVD degeneration. These strategies are designed to tailor the hallmarks of degenerative cascades within the disc microenvironment, for example in targeting loss of notochordal and progenitor phenotype, apoptosis and senescence results in lower cellularity, dysregulation of ECM, increase of inflammation, neovascularization, sensory nerve innervation, loss hydration, and mechanical-mediated degeneration and spine instability.

**Fig. 4:**
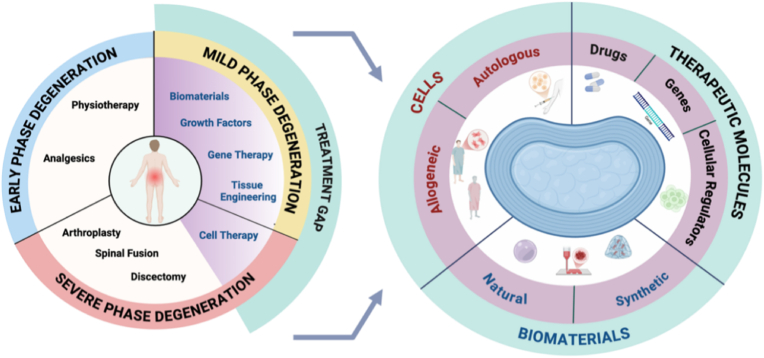
Schematics depicting the contemporary treatment gap for LBP associated with disc degeneration and showing the regenerative approach through precision medicine, tailoring to different IVD degeneration stages. Identification of the therapeutic molecules and development of functional biomaterials incorporation with cells tailored to mild and advanced stages of IVD degeneration ultimately improving treatment efficacy. Schematic was created by BioRender.

For cell-based therapy, for example, mesenchymal stem cells (MSCs), either autologous or allogeneic, pre-conditioned towards IVD-like cells, can be transplanted within biomaterial systems for IVD regeneration. These strategies offer higher efficacy due to cell transplantation in a cytoprotective environment, either natural or synthetic biomaterials, avoiding low cell retention and poor graft survival (Fig. 4) [51]. The regenerative approach utilising MSCs, notochordal or nucleus pulposus cells and IVD-derived progenitor cells aim to replenish the depleted notochordal and progenitor phenotype, and native disc cell population and ECM degradation, an indicative hallmark of severe stage of IVD degeneration. Transplanted cells secrete trophic factors that stimulate ECM synthesis to restore anabolic matrix production, counteract inflammatory mediators, and promote tissue homeostasis [52]. Gene therapy offers targeted modulation of key molecular pathways. By delivering genes that upregulate anabolic factors (e.g., SOX9, TGF-β1) or suppress catabolic enzymes and inflammatory cytokines (e.g., MMPs, IL-1β), gene therapy directly addresses the imbalanced metabolic environment within the degenerative disc [53].

For biomaterial platform, the acellular ECM-based scaffolds engineered as hydrogels and biomimetic multilayer nanofiber systems for NP and AF, respectively provide a three-dimensional matrix that mimics the native ECM is tailored for mild stage of IVD degeneration by providing conducive microenvironment to resident IVD cells to regulate their functions, thus maintaining ECM homeostasis. The design of biomaterials involves strategy as an advanced delivery system to deliver the cargo (therapeutic molecules and cells) to the host in a time-dependent manner. These include the delivery of cells, genes, regulators, growth factors, and drugs that can target the disease mechanism underlying IVD degeneration, including boosting the disc's anabolic phenotype while decreasing its catabolic, inflammatory, nociceptive and degenerative cascade at moderate to severe IVD degeneration [54]. Three dimensional (3D) bioprinting approach in biomaterial-based tissue engineering allows for the modulation of mechanical properties of the developed biomaterial (Table 3) constructs to maintain the required flexibility following implantation while also providing the stiffness needed to provide mechanical strength, thus support loads and stability throughout spine – effective for severe stage of IVD degeneration [55]. In combination, artificial intelligence (AI)-driven diagnostics integrate imaging, genomic, and clinical data to enable earlier detection of degeneration tailored to disease severity, which in line with next generation of patient-focused precision therapy for the treatment plan of degenerative disc disease [56].

Overall, regenerative strategies aim to address cell loss, matrix degradation, inflammation, and mechanical failure the key pathological hallmarks of IVD degeneration. These approaches represent a paradigm shift from symptomatic palliation toward true biological repair and functional restoration of the intervertebral disc.

### 6.1 Layered treatment strategies in regenerative therapy for intervertebral disc degeneration

#### 6.1.1 Mild degeneration: biomaterials regulating the microenvironment

In early-stage IVD degeneration, characterized by biochemical imbalances and microenvironmental disruption without structural collapse, biomaterial-based strategies aim to restore disc homeostasis. Hydrogels, such as methacrylated cellulose derivatives (e.g., CMC, MC) or alginate-pNIPAAm composites, are injected to mimic the nucleus pulposus (NP) and annulus fibrosus (AF). These materials regulate hypoxia, acidity, and inflammation by delivering anti-inflammatory agents (e.g., fucoidan) or growth factors (e.g., GDF-5) to suppress catabolic enzymes like MMP-3 and ADAMTS-4, while promoting ECM synthesis of aggrecan and collagen II [57]. For example, Mg^2+^-loaded hydrogels mitigate oxidative stress in NP cells, enhancing ECM regeneration under inflammatory conditions [58]. Such approaches delay degeneration by stabilizing the hypoxic microenvironment via HIF-1α signalling [59].

#### 6.1.2 Moderate to severe degeneration: cell-based therapy

In moderate IVD degeneration, where cellular loss and ECM degradation accelerate, incorporating mesenchymal stem cells (MSCs), notochordal or NP-like cells with scaffolds to repopulate the disc cellularity and ECM synthesis. Injectable hydrogels (e.g., GelMA-alginate microspheres) or nanofiber scaffolds deliver MSCs, which differentiate into NP-like cells under hypoxia and growth factor stimulation (e.g., TGF-β, BMP-2) [2]. Clinical trials, such as the EuroDISC study, demonstrate that autologous NP cell transplantation post-discectomy reduces pain and preserves disc height [2]. Similarly, MSC-laden scaffolds functionalized with SKP/RGD peptides recruit endogenous stem cells and enhance ECM production, addressing the cell-depleted microenvironment [58,59]. These composites outperform standalone biomaterials by synergizing cell survival and matrix synthesis [59].

#### 6.1.3 Severe degeneration: tissue-engineered disc replacements

For advanced IVD degeneration with structural failure, tissue-engineered discs replace damaged tissue. Engineered disc analogs (e.g., disc-like angle ply structures, DAPS) replicate NP-AF-endplate architecture using layered hydrogels (e.g., PEG-nanocellulose) and mechanically robust polymers (e.g., PCL). Preclinical models show that DAPS implants integrate with vertebral bone, restore compressive/tensile properties, and resist fibrosis over 20 weeks [60]. Composite scaffolds (e.g., alginate-cellulose matrices) loaded with dual growth factors (TGF-β/BMP-2) and MSC homing peptides enable biomimetic ECM regeneration, achieving functional disc height recovery [58]. These constructs are prioritized over fusion surgery to preserve spinal mobility [59].

## 7 Functional biomaterials for IVD regeneration

The intervertebral disc, comprising the NP and AF, requires materials that mimic its unique structure, ECM composition and mechanical properties. Hydrogels are widely used because of their capacity to downregulate the expression of catabolic markers [61], restore disc height [62], absorb mechanical loads [63], and act as a scaffold for cellular formation and regeneration [64]. They resemble the hydrated matrix of the NP. Moreover, composite materials that combine the natural ability of hydrogels with the robustness of fibres are being developed to reinforce the AF and NP and prevent further degradation. A primary objective of disc regeneration therapy is to reinstate the biomechanical functionality of the IVD, which supports the trunk and facilitates mobility, since the 10.13039/100006209NP aids in transmitting axial stress to the AF [59].

Advanced cell delivery systems are made possible by biomaterials, which provide creative means of regulating the location and timing of within the body. As referred to in Table 2, Table 3 in the biomaterial section, those polymers can be modified to give drugs in a prolong manner or to focus on certain sites of direct in situ cell release. Such an approach offers an attractive solution to the treatment of intervertebral disc degeneration, with the potential for targeted repair that can directly regenerate damaged disc tissue, whilst preserving the structure and function of adjacent healthy spinal elements [65]. The most robust delivery systems and biomolecules to incorporate within these biofunctional materials are likely to protect both stem cells, as well as tissue-specific cells made from stem cells against immunological assault, hypoxic conditions, and stress responding to transplantation long-term [66].

**Table 2:** The use of biomaterials, therapeutic molecules of antioxidants and growth factors in IVD degeneration.

Biomaterials	Model	Findings	Refs
HA hydrogel	Rabbit	Disc height was increased at weeks four and eight	[67]
Chitosan	Human	produced collagen type II and aggrecan and remained viable up to 70 % after 4 weeks in chitosanglycerol	[67]
Agarose	Human	Hybrid scaffold (NP + AF) for *in vitro* testing.	[68]
Fibrin	porcine	Suppression of the acute proinflammatory cytokine (TNF-, IL-1 , IL-6) production	[69]
Collagen	Rats	Promotion of the formation of cell aggregative spheroids that facilitate the maintenance of the original disc NP phenotype, upregulation of the expression of chondrogenic genes	[70]
Alginate	(Rabbit, Sheep)	Induction of endogenous NP cells and NP progenitor cells (GD2Tie2 cells), leading to endogenous IVD repair	[71]

**Therapeutic molecules**			

**Antioxidants**			
NAC	Rats	Suppressing catabolic and proinflammatory phenotype induced by H_2_O_2_	[72]
RSV	Human	Suppressing apoptosis	[73]
Fullerol	Human	Retarding matrix catabolism induced by H2O2 Rabbit discs (intradiscal injection)	[74]
GSH	Human	Suppressing apoptosis and matrix catabolic phenotype induced by H_2_O_2_	[74]
**Growth factors**			
fibroblast growth factor (FGF)	human	Stimulate the proliferation of IVD cells and accumulation of ECM	[75]
(TGF-b)	human	support to drive *in vitro* chondrogenesis of human mesenchymal stem cells (hMSC)	[76]

**Table 3:** The mechanical properties and biocompatibility of hydrogels, nanofiber scaffolds, and 3D-printed materials, followed by examples of 3D-printed dual growth factor scaffolds used for nucleus pulposus and annulus fibrosus repair.

Feature	Hydrogels (e.g., HA)	Nanofiber Scaffolds	3D-Printed Materials
Mechanical Properties	Generally low mechanical strength and stiffness; soft and highly hydrated, mimicking native NP tissue elasticity but limited load-bearing capacity [77]	Moderate tensile strength and flexibility; high surface area and ECM-like fibrous structure; mechanical properties depend on polymer type and fiber alignment [78]	Wide range of tunable mechanical properties; can be designed for high stiffness and structural support; layer-by-layer fabrication allows precise control of scaffold architecture [79]
Biocompatibility	Excellent biocompatibility and bioactivity; supports cell viability and proliferation; often biodegradable and mimics native extracellular matrix (ECM) [77]	High biocompatibility; promotes cell adhesion, migration, and proliferation due to nanofiber morphology; can be functionalized for enhanced bioactivity [78].	Generally biocompatible depending on material used (e.g., methacrylated hydrogels, resins, PCL); post-processing can improve biocompatibility; can incorporate bioactive molecules [79].
Porosity & Cell Interaction	Highly porous, allowing nutrient diffusion but limited mechanical integrity; supports 3D cell encapsulation [77]	High porosity and interconnected pores; mimics ECM fibrous network enhancing cell infiltration and alignment [78].	Porosity and pore size can be precisely controlled; supports cell infiltration and vascularization; can be combined with nanofibers for hierarchical structures [79].

Biomaterials regulate the IVD microenvironment primarily by targeting oxidative stress and inflammatory processes that drive degeneration. They can scavenge reactive oxygen species (ROS), thereby reducing oxidative damage and attenuating inflammation within the extracellular matrix (ECM). Additionally, biomaterials function as delivery vehicles for anti-inflammatory agents and growth factors such as transforming growth factor-beta (TGF-β), which promote cell proliferation and ECM synthesis, thereby supporting tissue repair processes. They also inhibit the expression of matrix metalloproteinases (MMPs), enzymes responsible for ECM degradation, thus helping to preserve disc structural integrity. Some designed biomaterials further modulate immune responses by recruiting immune cells and promoting macrophage polarization toward the M2 phenotype, which facilitates repair and regeneration [80].

Furthermore, biomaterials facilitate targeted delivery of therapeutic molecules and enhance the migration and survival of endogenous stem or progenitor cells within the degenerative disc environment. These mechanisms collectively shift the inflammatory microenvironment toward a regenerative state, promoting tissue repair and slowing degeneration progression. Although these strategies show promise *in vitro* and in small animal studies, further validation in large animal models is essential before clinical translation. Overall, biomaterials actively remodel the disc's inflammatory and ECM landscape, fostering regeneration and improving disc health [80,81].

### 7.1 Biomaterials for cell delivery

For treatment of IVD degeneration, the hydrogels including hyaluronic acid (HA) hydrogel could be utilised as a delivery system to administer therapeutic agents including cells incorporate into IVD thorough injection. For example, Liu et al. employed HA hydrogel to deliver M2c macrophages in the degenerated caudal discs of rats. They demonstrated that M2c-Exoss indirectly facilitated the phosphorylation of Smad3 and augmented the transduction of the TGF-β pathway by suppressing the expression of CILP protein in disc degeneration [82]. Combination of biomaterials such as HA and collagen were made possible to further improve the functionality of the hydrogel as well as its therapeutic effects. For example, type II collagen enriched with HA hydrogel in an *in vitro* study by Isa et al. replicates the NP microenvironment, guiding hWJ-MSCs towards the NP phenotype [83].

Another study uses a blend of agarose and collagen as a transport medium for bovine NP cells which is shown to enhance the production of GAG, increased cell adhesion and FAK activation which are key for integrin-mediated mechanotransduction mechanisms [84]. Furthermore, due to its high biocompatibility, degradability as well as low immunogenicity, chitosan is a promising biomaterial for the treatment of IVD degeneration. A hydrogel with a 5:3:2 ratio of chitosan, 10.13039/100016376HA, and kartogenin demonstrated optimal swelling properties, supported adipose-derived stem cell proliferation and differentiation into nucleus pulposus-like cells, and exhibited mechanical properties similar to native nucleus pulposus tissue [85].

### 7.2 Therapeutic biomaterials

As IVD degeneration advances, the loss of proteoglycans in the NP diminishes its swelling pressure, compromises the mechanical characteristics of the extracellular matrix [86], and results in reduced flexibility and annulus fibrosus delamination [87], rendering the gelatinous nucleus pulposus an attractive target for therapeutic strategies utilising hydrogels and other soft biomaterials.

For anti-inflammatory biomaterials, they can regulate the inflammatory microenvironment to repair degenerated intervertebral discs. By having to control the regulation of the inflammatory microenvironment using biomaterials, the IVD can be induced to improve in self-repair. Targeting anti-inflammatory factors and utilising advanced biomaterials for controlled release of growth factors like TGF-β and connective tissue growth factor (CTGF) to promote ECM deposition in regenerating intervertebral discs, offering promising therapeutic avenues for alleviating intervertebral disc degeneration. Hyaluronic acid (HA) molecules usually interact with N-terminals proteoglycans in the extracellular matrix [88], which are essential for modulating inflammatory signalling. HA is known to exhibit anti-inflammatory effect. Inoue et al. found that injecting a HA hydrogel in a rabbit model of IVD degeneration increased disc height, reduced inflammation (including IL-6), and slowed disc degeneration by promoting tissue hydration repair [89]. We demonstrated that possible mechanism of HA hydrogel exerted anti-inflammatory effects by interfering the binding of IL-1β and IL-6 to their respective receptors, thereby inhibiting the transcription of genes encoding proinflammatory cytokines mediating acute-phase signalling [85]. Lowering the levels of inflammatory factors can also help IVD repair, since these factors can speed up IVD degeneration. A study by Smith et al. increases long term inhibitory of IL-1β by combining poly (lactic-co-glycolic acid) (PLGA) and IL-1 receptor antagonist (IL-1ra) to create PLGA microparticles by 35 days via *in vitro* study and 7 days via in vivo study [90].

Among biomaterials with anti-nociceptive properties, HA has emerged as a promising candidate for alleviating nociceptive pain. In our study, HA hydrogel demonstrated robust efficacy in reducing thermal hyperalgesia and mechanical allodynia. This anti-nociceptive effect was associated with modulation of the ascending pain pathway through downregulation of nociceptive markers such as c-Fos and substance P in the dorsal horn of the spinal cord, thereby suppressing central pain processing. Additionally, HA hydrogel inhibited nociceptive sensory nerve innervation within the aneural IVD following implantation in a rat tail disc injury model, possibly through the NGF molecule, a key neurogenic mediator involved in regulating neurite ingrowth [86].

**Fig. 5:**
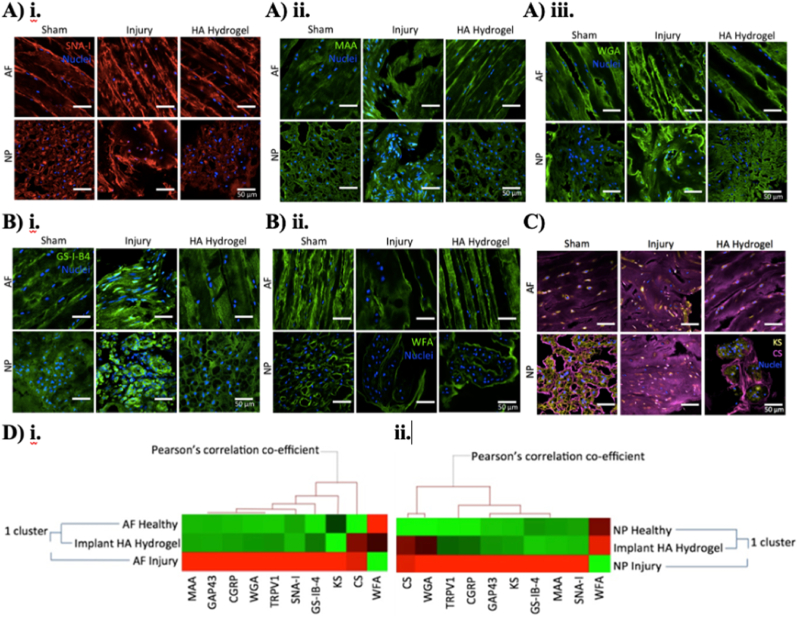
Effects of HA-hydrogel implantation on glycosylation in the injury-induced pain model; (A–C) Assessment of glycosylation on day 29 after injury through quantification of lectin binding and glycosaminoglycan expression (A.i-iii) SNA-I (red label), MAA (green label) and WGA (green label) binding to α-(2,6)-linked sialic acid, α-(2,3) sialylated galactose and N-acetyl-D-glucosamine or sialic acid was observed in the sham control, injury and HA-hydrogel-treated injury groups respectively, in annulus fibrosus (AF) and nucleus pulposus (NP) tissues. (B.i-ii) GS-I-B4 (green label) and WFA (green label) binding to α-galactose and terminal GalNAc motifs was observed in the untreated injury group in AF and NP tissues of sham control, untreated injury and HA-hydrogel-treated injury groups. (C) Expressions of chondroitin sulfate (purple label) and keratan sulfate (yellow label) were denoted in the sham control, untreated injury and HA-hydrogel-treated injury groups, in AF and NP tissues. (D) Correlation between glycoprofiles and nociception markers. Clustering analysis was carried out on the quantification profiles from confocal fluorescence microscopy of glycosylation and sensory hyper-innervation and nociceptive markers in AF (D.i) and NP (D.ii) tissues for the sham control, untreated injury and HA-hydrogel-treated injury groups. (n = 4). Scale bar = 50 μm. Figure adaptation from Mohd Isa et al., 2018 [92].

ECM repair-mediated hydrogels are favourable for mimicking the native ECM composition of the IVD in facilitating tissue repair. HA, a predominant ECM component in the NP. 10.13039/100016376HA hydrogel has demonstrated the ability to promote ECM deposition and support tissue repair through pathways involving transforming growth factor-beta 1 (TGF-β1) and Smad3 signaling. Additionally, it has been shown to modulate the glycan signature in both the AF and NP by downregulating sialylation and galactosylation, thereby contributing to tissue regeneration [86] (Fig. 5). HA also enhanced T1ρ mapping signals, as indicated by MRI, reflecting increased proteoglycan deposition eight weeks after hydrogel injection. At the tissue level, we observed homogeneous distribution and higher cellularity in the 10.13039/100006209NP and well-organised AF lamellae, underpinning the therapeutic potential of ECM-based hydrogels in supporting structural restoration [89]. Collagen, abundantly present in the AF, has been developed in hydrogel format that promote ECM-mediated tissue repair. For example, an *in vitro* study by Du et al. demonstrated that pretreated TGF-β1 AF encapsulated in collagen hydrogel on polyurethane membranes showed an enhanced gene and protein expressions which may be a suitable biomaterial for the repair of AF ruptures [91]. Low adhesive scaffold collagen promoted the development of cell aggregative spheroids in rats, preserving the initial NP phenotype and upregulating chondrogenic genes [70].

Mechanical support scaffolds represent a key strategy for replicating the mechanical properties of the IVD, providing structural reinforcement. Sun et al. developed mechanically support IVD scaffold by covalently attaching TGF-β3 and CTGF to the surface of polydopamine nanoparticles, loading with bone marrow-derived mesenchymal stem cells (BM-MSCs) in a 3D-printed polycaprolactone framework that could release two growth factors to promote deposition of type II collagen and glycosaminoglycan in the 10.13039/100006209NP zone, and type I collagen in the AF zone. The dual-GFs/MSCs/IVD scaffold exhibited a higher compressive Young's modulus that closely mimicked native IVD tissue, thereby enhancing shock absorption and enabling the reconstructed IVD to withstand mechanical loading [93]. A study by Buser et al. also concludes that fibrin sealant promoted structural, compositional, and mechanical repair of surgically damaged intervertebral discs by inhibiting nuclear fibrosis, enhancing proteoglycan recovery, reducing TNF-α while upregulating IL-4 and TGF-β, and restoring stiffness and pressure resistance to control levels within 6–12 weeks post-treatment [69].

Protein solutions enable cell development and anabolic responses to be injected into the IVD in an attempt to temporarily halt degeneration and prevent future disc degeneration [94]. It has been previously shown that external growth factors influence the IVD [95,96]. Specific growth factors that induce bone and cartilage formation are bone morphogenic proteins (OP-1, BMP-14) and members of the transforming growth factor-β. *In vivo* rabbit study showed that the injection of OP-1 in intervertebral disc resulted in increased proteoglycan content of the nucleus pulposus and elevation of disc height. Imai et al. This study has been repeated and shown to improve MRI appearances of disc degeneration [97,98]. Previous work including rat studies have shown that the injection of OP-1 into the IVD leads to an anabolic response that restores normal disc architecture [99]. Research using sheep models of IVDD has demonstrated that injecting BMP-13 can prevent the loss of hydration in the NP, as indicated by Wei et al., in 2009. Injection of BMP-2 in a rabbit model has been found to worsen the degeneration of the IVD, showing negative effects of therapeutic proteins. The efficacy of protein injections is limited by their short-term therapeutic effects. Enhancing this promising therapy for disc degeneration might involve creating a slow-release carrier or gene-based delivery system to prolong the therapeutic effects**.**

In conclusion, several examples illustrate that the advancement of biomaterials capable of replicating the distinctive structure and mechanical properties of the intervertebral disc, regulating inflammation, presents significant potential for enhancing the repair and regeneration of degenerated intervertebral discs.

### 7.3 Biomaterials for IVD tissue engineering

Tissue engineering is an interdisciplinary discipline that integrates biology, engineering, and medicine to provide techniques for cultivating and restoring tissues or organs within the human body. When the intervertebral disc shows extensive degeneration and significant loss of cellularity, the damage is unlikely to be reversed with cell-based implantation or therapeutic protein injections alone. Functional replacements for injured disc tissues need to be inserted as a scaffold, and cells should be physically conditioned by mechanical or electrical stimulation [[80], [81], [82]].

Biomimetic NP and AF scaffolds are used to replace the severely degenerated disc. For example, multilayered angle-ply scaffold, consisting of concentric layers of lamellar sheets using silk fibroin been engineered to mimic the native architecture and mechanical behaviour of the AF, thereby facilitating guided cellular alignment and promoting cell proliferation, differentiation, and ECM deposition of sulfated glycosaminoglycan and collagen type I, indicating a favourable environment for AF and MSC-derived AF-like cells [103]. Tissue engineering advancements have allowed the creation of tissue-engineered entire implanted intervertebral disc. The TE-IVD was shown to integrate into the disc area in a rat tail model and had comparable characteristics to the natural disc in biomechanical and biochemical assessments [104,105]. In the study, total disc replacement (TDR) was conducted in the canine cervical spine, where the TE-IVDs successfully integrated with the host tissue and partially preserved disc height. When TE-IVDs are used with protein and gene-based treatments, further clinical enhancements are observed. In a study by H. Xin et al. (2013), a canine model of total disc replacement (TDR) using tissue-engineered intervertebral discs (TEIVDs) including the human telomerase reverse transcriptase (hTERT) gene in nucleus pulposus (NP) cells demonstrated an anti-degenerative effect in the group with hTERT [106].

## 8 Cell-based therapy

Cell therapy has grown in favour of a regenerative strategy [52,107,108]. These include the use of chondrocytes, NP, notochordal or IVD progenitor cells, and mesenchymal stem cells (MSCs) include induced pluripotent stem cells (iPSCs), adipose-derived mesenchymal stem cells (ADSCs), bone marrow-derived mesenchymal stem cells (BM-MSCs) and Wharton-Jelly derived (WJ-MSCs) for IVD regeneration (Table 4).

**Table 4:** Regenerative strategies of IVD include the use of NP and various sources of stem cells.

Cell-based therapy	Model	Finding	Refs
NP tissue	Rats	8 weeks; delays degeneration compared with sham (no cells)	[109]
NP + AF cells	Rats	33 weeks; good integration up to 8 months	[110]
Human NP cells	Rabbit	24 weeks; good IVD height, histology, aggrecan and type II collagen expression	[111]
NP cells	Rabbit	24 weeks; delayed degeneration, maintained disc height; improvements in ECM production, segmental instability and T2-weighted MRI signal intensity	[112]
hUTC	Rabbit	12 weeks; hUTC in hydrogel might help restore MRI, biomechanics and histology	[113]
BMSCs	Rabbit	10 weeks; quantitative and noninvasive T2-weighted MRI mapping could be used to evaluate NP regeneration	[114]
BMSCs	Rabbit	8 weeks; percutaneous delivery and HyStem® augment NP regeneration	[115]
ADSCs			
WJ-MSCs			
**Gene therapy**	**Model**	**Finding**	**Refs**

Virus-mediated Adenovirus Target. SOX-9, GFP	Human disc NP cell	The AdSox9 virus efficiently transduced HTB-94 cells and degenerated human disc cells increased Sox9 production and Type 2 collagen production.	[116]
Non-virus-mediated Microbubble-enhanced ultrasound	Rat disc NP cell	Ultrasound transfection method with microbubbles significantly enhanced the transfection efficiency of plasmid DNA into the nucleus pulposus cells in vivo.	[117]
CRISPR Cas9	Human disc NP cells	Successfully transduce hNPCs and downregulate TNFR1/IL1R1 expression	[118]

Abbreviations: AF, annulus fibrosus; BMSCs, bone marrow-derived stem cell; hUTC, human umbilical cord tissue; HA hydrogel, hyaluronic acid (HA) hydrogel; NAC, N-Acetylcysteine; RSV, Resveratrol; GSH, Glutathione; SOX-9, transcription factor 9; TIMP-1, tissue inhibitor of metalloproteinase; IGF-1, insulin-like growth factor-1; TGF-b, transforming growth factor-beta; GFP, growth and differentiation factor; TNF, tumor necrosis factor; Cas9, CRISPR-associated protein 9; CRISPR, Clustered Regularly Interspaced Short Palindromic Repeats; IL, interleukin.

MSCs are multipotent non-hematopoietic stem cells isolated from several sources, for instance, adipose tissue, umbilical cord, bone marrow, placenta, amniotic fluid, fat, tooth pulp, etc [119,120]. They can differentiate into various mesenchymal tissue types such as the chondrogenic lineage and IVD-cell-specific phenotypes. In *in vitro* cultures, MSCs can differentiate into adipocytes, osteoblasts and chondroblasts [121,122]. Both CD86, CD40, and CD80 as well as the major histocompatibility complex-II (MHC-II) are absent in MSCs due to its immuno-privileged properties. The MSCs then respond to a variety of conditions, notably growth factors and oxygen levels, and differentiate into IVD cells. For these reasons, MSCs are a viable candidate for intervertebral disc repair as they can be easily obtained, and subsequent to differentiation, can deposit proteoglycans and collagen for the disc extracellular matrix.

Currently MSCs are tested to be safe to use and have tremendous clinical significance in the field of regenerative medicine [123]. The US FDA has approved over 60 MSC clinical trials, with the primary areas of interest being hematopoietic stem cell transplantation, autoimmune illnesses, gene therapy vectors, and tissue repair [124]. However, around 1000 clinical trials involving MSCs for the treatment of different disorders have been launched globally. Given the encouraging findings of employing MSCs in many illness states, many researchers are interested in studying MSCs in the treatment of IVDD [125]. We summarise the harvesting difficulty, differentiation potential, and immunogenicity between MSC Table 5.

**Table 5:** Comparison of stem cell sources based on harvesting difficulty, differentiation potential, and immunogenicity.

MSC Source	Harvesting Difficulty	Differentiation Efficiency	Immunogenicity	Refs
**BM-MSCs** (Bone Marrow)	Invasive (bone marrow aspiration); low cell yield (∼0.001–0.01 % of mononuclear cells); donor site morbidity	High osteogenic and chondrogenic; moderate adipogenic potential	Low expression of MHC I; negligible MHC II; moderate immunomodulatory capacity	[126]
**ADSCs** (Adipose-Derived)	Minimally invasive (liposuction); high yield (∼500 × more than BM-MSCs); donor-dependent variability	High adipogenic; moderate osteogenic and chondrogenic differentiation	Similar immunophenotype to BM-MSCs; strong immunosuppressive effects	[127]
**WJ-MSCs** (Wharton's Jelly)	Non-invasive (postnatal umbilical cord); ethically acceptable; abundant and young cells	High chondrogenic and neurogenic; variable osteogenic potential	Very low immunogenicity; do not express MHC II or co-stimulatory molecules (CD80/CD86); suitable for allogeneic use	[128,129]
**iPSCs** (Induced Pluripotent)	Minimally invasive; Reprogrammed from somatic cells (e.g., skin fibroblasts, peripheral blood); technically demanding and costly	Pluripotent; can differentiate into all three germ layers, including NP- and NC-like cells	Low immunogenicity if autologous; potential immune rejection if allogeneic; risk of teratoma formation	[130]

### 8.1 Chondrocytes, notochordal and IVD progenitor cells

As intervertebral disc degeneration develops, the efficacy of therapeutic protein injections and gene-based therapies decreases because there are less cells in the intervertebral disc to respond to these signals. Cell-based therapy is a successful treatment for mid-stage degeneration that increases the number of IVD cells [131]. Research has shown that both autologous and allogenic disc cells may survive in the disc. An investigation with an injured canine model showed that implanting NP helped rebuild the ECM and prevented further disc degradation. Studies on pigs have revealed that articular chondrocytes are more successful than MSCs in mending discs, indicating that these specialized cells are more likely to survive the disc's low-oxygen environment [132].

Recent studies have underscored the potential of notochordal cells (NCs), particularly CD24-positive nucleus pulposus (NP) progenitors, in the regeneration of IVDs. These cells have been identified as crucial players in halting and reversing disc degeneration. Research indicates that CD24-positive NP cells exhibit robust multipotent differentiation capabilities and self-renewal potential *in vitro*, alongside abundant expression of key markers such as brachyury, SHH, and GLUT-1, indicative of their notochordal lineage [133]. Notochordal cells secrete factors that support IVD health by promoting regeneration, reducing inflammation, guiding stem cell differentiation, and preserving extracellular matrix integrity. These findings highlight their key role in maintaining and potentially repairing IVD tissue [134]. Transplantation experiments have demonstrated their efficacy in restoring degenerate discs, evidenced by increased disc height, restored MRI T2-weighted signal intensity, and NP structure. Mechanistically, activation of the HIF-1α–Notch1 pathway appears pivotal in maintaining the phenotypic characteristics and regenerative capacity of CD24-positive NP cells. These findings highlight CD24-positive NP cells as promising candidates for cell-based therapies aimed at addressing disc degeneration, suggesting a path forward in clinical applications for treating spinal disc disorders [135].

IVD progenitor cells (IVDSPCs) can be harvested autologously during disc herniation procedures, minimizing immune rejection and ethical concerns. These endogenous stem cells, which resemble mesenchymal stem cells in surface markers, proliferation, and differentiation capacity, offer the unique advantage of being naturally adapted to the harsh, avascular microenvironment of the IVD. Despite their potential, the biological properties of IVDSPCs—particularly subtypes like nucleus pulposus stem/progenitor cells (NPSPCs), annulus fibrosus stem/progenitor cells (AFSPCs), remain insufficiently characterised, especially under in vivo conditions. Therefore, future research should focus on accurate identification and characterization of these cells using advanced technologies and animal models. Enhancing endogenous repair by promoting the migration, activation, and survival of native IVDSPCs within degenerated discs is a key therapeutic goal [136].

### 8.2 Induced pluripotent stem cells (iPSCs)

Induced pluripotent stem cells (iPSCs) are somatic cells that have been genetically reprogrammed to express specific genes and factors, differentiating them to a phenotype to embryonic stem cells. iPSCs offer potential for IVD regeneration by serving as a cell source that can be differentiated into NP and notochordal-like cells, essential for IVD formation and homeostasis.

For example, human iPSCs were derived from human neonatal foreskin fibroblasts using episomal plasmids carrying reprogramming genes of Oct4, Sox2, Nanog, Lin28, L-Myc, Klf4, and SV40LT have been shown to differentiated into NP-like cells, expressing positive pluripotent marker expression of TRA1–60, SOX2, OCT4, TRA1–81, and SSEA-4 *in vitro* [137]. Human iPSCs treated with GSK3i exhibited upregulation of primitive streak mesoderm (PSM) markers (brachyury, MIXL1, FOXF1) and downregulation of pluripotency markers (Nanog, Oct4, Sox2), while retaining notochordal (NC) phenotype markers for up to 8 weeks *in vitro*. At 8 weeks post-injection, iPSC-derived NC-conditioned media (iNC-CM) maintained the expression of notochordal markers (Keratin 18, Keratin 19, Noto, and brachyury) following annular puncture-induced intervertebral disc degeneration in a porcine model [20].

Zhang et al. established a protocol for the *in vitro* differentiation of human iPSCs into notochord-like (NCLs) and nucleus pulposus (NP)-like cells. They used the CRISPR/Cas9 technique to knock in the enhanced green fluorescent protein (EGFP) gene at the stop codon of NOTO to monitor differentiation into NCLs, followed by further differentiation in the presence of TGF-β3 towards NP-like cells. These *in vitro*-derived NCLs resemble adolescent human NP cells, expressing brachyury and FOXF2. Notably, TGF-β3-treated NCLs expressed TEK receptor tyrosine kinase (TIE2) and disialoganglioside 2 (GD2), which are surface markers indicative of disc NP progenitors. In a puncture-induced rat tail disc injury model, the transplanted cells recovered disc height index (DHI), preserved of the NP cell population and promoted ECM deposition, as shown by type II collagen and aggrecan expression, suggesting that treatment of hiPSC-derived NP-likes facilitate NP regeneration and attenuate injury-induced IVD degeneration in rat tail model [138].

Conclusively, iPSC-derived notochordal cells not only maintain a stable phenotype *in vitro* and in vivo, but also demonstrate functional properties, and offer protective effects against disc degeneration—highlighting their therapeutic potential for intervertebral disc regeneration.

### 8.3 Adipose-derived mesenchymal stem cells

Adipose-derived stem cells (ADSCs) have the capacity to create intervertebral disc tissue. MSCs have typically outperformed differentiated disc cells in rebuilding disc shape following injection, both in laboratory settings and in real beings, despite some contradictory results [139]. Previous investigations in rabbit models found similar results when comparing the two cell lineages. MSCs are a greater and more accessible therapeutic alternative than IVD cells, making them an excellent replacement. Further investigation reveals that combining these two cell lines increases the function and lifespan of transplanted cells. There is a lot of hope that both cell types will help to repair the damaged disc [140].

Several preclinical research utilising various animal models have studied the utility of adult MSCs for treating IVDD during the last few decades. MSCs transplantation restores equilibrium to the IVD's damaged environment, which yields beneficial effects [141]. Furthermore, MSCs have the capacity to develop into NP-like cells and exhibit anti-catabolic and immunomodulatory functions [142]. Surprisingly, recent research has shown that MSCs and NPCs interact with trophic factors to allow NPC proliferation and protection in the degenerated disc by increasing the expression of NP markers such as SOX9, type II collagen, and proteoglycans, promoting ECM synthesis, and decreasing pro-inflammatory cytokines [143].

Similarly, several preclinical studies have examined how MSC paracrine activity enhances IVD regeneration [[109], [110], [111]]. When paracrine interactions in a co-culture system were investigated after obtaining MSCs, AF, and NPCs from the same donor, increased mRNA expression of ECM genes such as SOX9, collagen type 2 (COL2A1), and aggrecan (ACAN) were observed, as well as downregulation of MMPs, ADAMPTS, and pro-inflammatory factors, compared to monocultures [147].

### 8.4 Bone marrow-derived mesenchymal stem cells

**Fig. 6:**
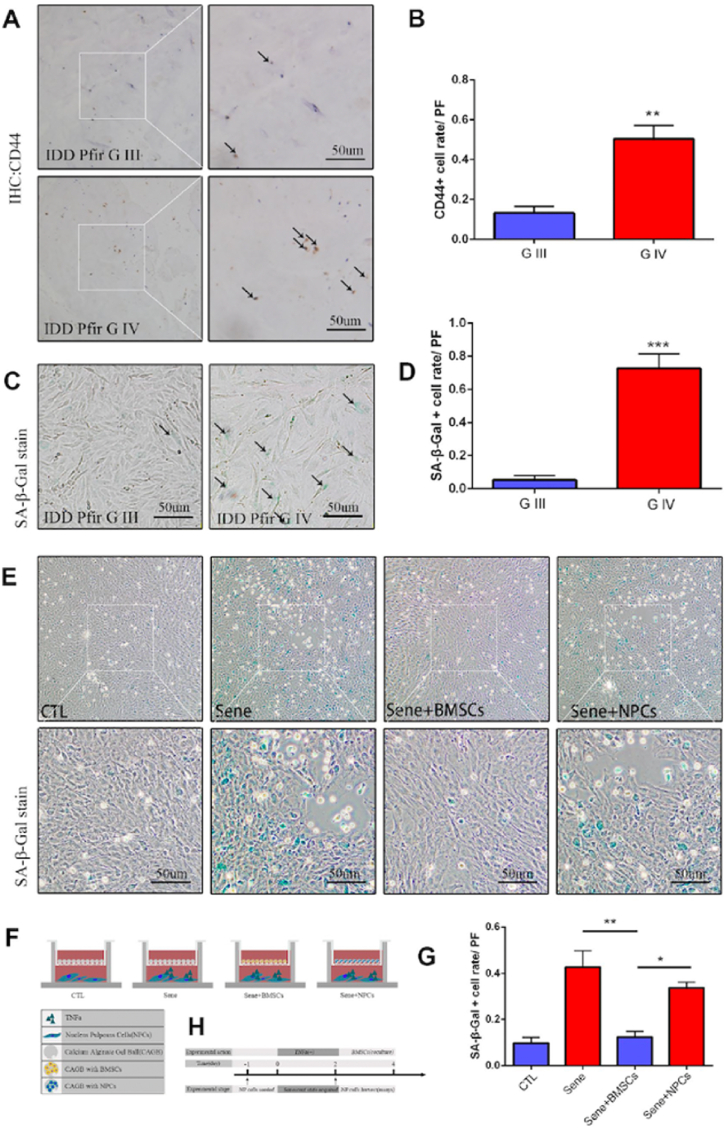
Mesenchymal stem cells activated in IDD, and cocultured BMSCs can alleviated the nucleus pulposus cells senescent rate *in vitro*. (A) Immunohistochemistry staining of CD44 in intervertebral disc degeneration of Pfirrman grade III (G III) and grade IV (G IV). n = 5, Scale bar, 50um. (B) CD44 positive cell rate between G III and G IV were determined by using Image J software. (C) SA-βGal staining of primary NP cells of IDD Pfirrman grade IV and Pfirrman grade III. n = 5, Scale bar, 50um. (D) SA-β-Gal positive cell rate between G III and G IV were determined by using Image J software. (E) 3D coculture models of were established as schematic diagram described. SA-βGal staining of senescent NP cells after 2 days coculture of normal NP cells + blank calcium alginate gel balls (CTL), senescent NP cells + blank calcium alginate gel balls (Sene), senescent NP cells + calcium alginate gel balls with BMSCs (Sene + BMSCs), senescent NP cells + calcium alginate gel balls with normal NP cells(Sene + NPCs), n = 3, Scale bar, 50um. (F) Co-cultivation pattern design and experimental groups. (G) SAβ-Gal positive cell rate between CTL, Sene, Sene + BMSCs and Sene + NPCs groups were determined by using Image J software. (H) Experimental flow diagram Values represent means ± S.D. Significant differences between different groups are indicated as ∗P < 0.05, ∗∗P < 0.01, ∗∗∗P < 0.001. PF: per field. Figure adaptation from Ref. [148].

Upregulation of NPC proliferation, elevated type II collagen yield, downregulation of MMP-9 expression, and decreased TGF-β and nuclear factor-κB (NF-κB) signalling were discovered in pre-senescent NPCs in co-culture system of TNF-α-induced degradation of NPC through BM-MSCs (Fig. 6) [148]. It was phase I/II clinical research to evaluate the feasibility and subjective clinical efficacy of promoting repair of degenerated intervertebral discs (IVD) in patients with degenerative IVD, using autologous MSCs implanted in tricalcium phosphate, as means to overcome the need of surgical bone transplantation. The data revealed that 80 % of the patients underwent lumbar fusion with no adverse consequences. Similarly, autologous BM-MSCs were injected into the NP region of ten IVDD patients in a pilot study without the use of a cell carrier. After a year, the patients' LBP and disability levels had improved [149] Furthermore, T2-weighted MRI demonstrated an increase in water content, although disc height restoration was not visible. Two female IVDD patients experienced identical results two years after receiving autologous BM-MSCs with 20 collagen-porous sponge injections [150].

### 8.5 Wharton Jelly-derived mesenchymal stem cells

Autologous MSCs from Wharton jelly of umbilical cord extracts are favourable since they are patient-matched, avoiding immuno-rejection concerns. Wharton Jelly-derived mesenchymal stem cells (WJ-MSCs) represent a readily accessible and ethically non-contentious source of stem cells. They exhibit high proliferative capacity, low immunogenicity, and strong immunomodulatory and anti-inflammatory properties, making them highly suitable for regenerative medicine applications. Due to the hypoimmunogenic nature of WJ-MSCs, allogeneic WJ-MSCs or donor umbilical cord extracts may be useful as readily accessible cells in IVD applications [130].

Currently, the majority of cord blood banks discard and do not freeze umbilical cord blood samples that include low hematopoietic stem cell numbers. Storing human Wharton jelly of umbilical cord extracts preserve MSCs that can be beneficial for MSC therapy for IVD degeneration. WJ-MSCs demonstrate robust proliferative capacity, multipotent differentiation potential, and the ability to secrete key ECM components, including collagen and proteoglycans, which are essential for ECM-mediating IVD repair and regeneration. A recent study reported that decellularised WJ matrix promoted cell migration, expression of collagen type 2, aggrecan, Sox9 and FOXO3a, anti-inflammatory action, and expression of progenitor/notochordal cells (CD24 positive cells) in vivo and *ex vivo* [151]. Decellularised WJ matrix promoted IVD phenotype by enhancing cell viability and upregulating key regulators of disc homeostasis, including SOX2, SOX9, and TRPS1 [152].

We demonstrated that human WJ-MSCs in collagen-based hydrogel can be differentiated into NP-like cells through TGF-β3 pathway with higher expression of NP-specific marker suggests a favourable microenvironment for IVD repair, implying the feasibility of WJ-MSCs transplantation in IVD degeneration [83]. WJ-MSCs exhibited anti-apoptosis by attenuating caspase-3 and Bax and inhibiting the activation of Wnt/β-catenin signalling in the compression-induced apoptosis in NPCs *in vitro* [153]. Human WJ-MSCs from different donors exhibited heterogeneity expression of TβRI/ALK5 and TβRII, with consistently high viability *in vitro*. In a rabbit lumbar disc puncture model, transplantation of high-dose hWJ-MSCs combined with Tissuefill® (HA derivatives) significantly restored disc water content, preserved disc structure, and reduced histological degeneration scores. These therapeutic effects were mediated by paracrine pathways [154].

The translational value of WJ-MSC as a viable and ethically accessible cell source for cell-based therapies in degenerative disc disease. Current findings on in vivo transplantation studies and development of WJ-MSC–based support the future regenerative strategies for treating disc degeneration.

## 9 Mesenchymal stem cell-derived exosomes

**Fig. 7:**
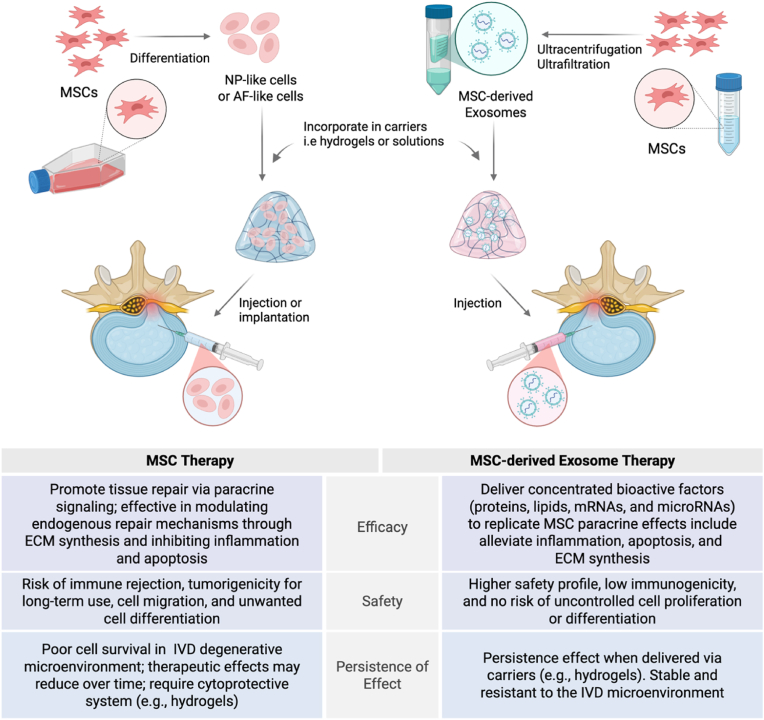
Comparison diagram of MSC and MSC-derived exosome therapies for IVD degeneration. MSC therapy involves cell differentiation into IVD-like cells, whereas exosomes are isolated from MSCs using methods such as ultracentrifugation and ultrafiltration. Both MSC-derived cells and exosomes can be incorporated into hydrogel systems for delivery into the IVD. Their efficacy, safety, and persistence of effect are summarized. Schematic was created by BioRender.

MSCs secretes paracrine, including number of bioactive factors such as platelet-derived growth factor (PDGF), matrix metalloproteinase-9 (MMP-9), matrix metalloproteinase-2 (MMP-2), interleukin-6 (IL-6), and insulin-like growth factor-1 (IGF-1) during their interaction with a compatible host. This product of MSCs exhibit potent regenerative, anti-inflammatory, anti-apoptotic, anti-fibrotic, and immunomodulatory effects. Consequently, they can repair tissue damage and control cellular immunity [155,156]. As such, MSCs-derived exosomes have emerged as a promising therapeutic approach for IVDD treatment. These nano-sized vesicles (30–100 nm) contain various bioactive molecules, including proteins, nucleic acids, and lipids, that can influence recipient cells [157]. MSCs produce more exosomes than other primary cells, with proteomic analyses showing 1927 different proteins in MSCs' exosomes that serve multiple functions necessary for their therapeutic effects [158,159]. We illustrate the fundamental approach MSCs-derived exosomes in comparison to MSC therapy in IVD degeneration, including method preparation, safety, efficacy and persistence effect (Fig. 7).

### 9.1 Therapeutic benefits of MSC‐derived exosomes

The therapeutic benefits of MSCs-derived exosomes in IVD degeneration treatment work through several mechanisms. They help restore extracellular matrix homeostasis by enhancing the expression of matrix-related molecules while inhibiting their decomposition [[126], [127], [128], [129]]. They promote cell proliferation and reduce apoptosis in nucleus pulposus cells, as demonstrated in various studies using different biochemical challenges such as IL-1β, TNF-α, and high glucose conditions [158,164]. Additionally, MSCs-derived exosomes exhibit significant anti-oxidative and anti-inflammatory effects. They can attenuate oxidative stress damage and protect against mitochondrial dysfunction [165]. They also show anti-inflammatory effects by inhibiting the production of inflammatory markers and the activation of the NLRP3 inflammasome [159].

To ensure long term delivery of exosome to the target site, hydrogels serve as an effective delivery platform for exosomes, enabling sustained release and localized retention, thereby enhancing their therapeutic efficacy in promoting intervertebral disc regeneration. For example, an exosome-loaded methacrylated silk fibroin hydrogel provided the sustained release of Cavin-2-engineered exosomes (M-EXO), which upregulated Dickkopf WNT signaling pathway inhibitor 2 to suppress the Wnt/β-catenin signaling pathway and activated the mitochondrial unfolded protein response. This dual modulation effectively reduces apoptosis and cellular senescence in both rat and human NP cells [166]. Thermosensitive acellular extracellular matrix (ECM) hydrogel loaded with ADSC-derived exosomes demonstrated an excellent sustained-release ability of the exosomes up to 28 days with higher NP cell viability was observed. This system regulated ECM metabolism by regulating matrix metalloproteinases (MMP-13) and inhibited pyroptosis by mitigating the inflammatory response *in vitro* [167].

However, several challenges need to be addressed before clinical implementation. These include the need for standardized isolation and characterization methods, optimal dosing and administration routes, and better understanding of therapeutic mechanisms [168]. Despite these challenges, MSCs-derived exosomes offer several advantages over direct cell therapy, including lower risk of tumour formation, better stability, and ability to cross biological barriers [161]. The future development of this therapeutic approach requires addressing these technical challenges and conducting more clinical trials. As concluded in the article, while MSCs-derived exosomes show remarkable potential for IVDD treatment, further research is needed to optimize their therapeutic application and understand their mechanisms of action more comprehensively [160].

### 9.2 Mechanisms of MSC‐derived exosomes

Mechanisms by which mesenchymal stem cell-derived exosomes (MSCs-exos) exert their therapeutic effects primarily involve the delivery of their bioactive cargo of lipids, proteins, and microRNAs (miRNAs) to target cells. These exosomes can modulate immune responses, promote tissue repair, and inhibit inflammatory processes through several pathways, including immunomodulation and cell internalisation through lipid and protein signalling to facilitate cellular functions, thus exhibiting their therapeutic effects.

MSCs-exos contain molecules such as miR-146a, which can inhibit pro-inflammatory signalling pathways. For instance, miR-146a delivered via MSCs-exos inhibits TRAF6 and IRAK1 in inflammatory macrophages, leading to decreased NF-κB activation and reduced cytokine production [169]. They suppress effector functions of inflammatory macrophages by downregulating cytokines like TNF-α, IL-1β, and IL-6, and promote the expansion of regulatory T cells (Tregs), contributing to immune tolerance and reduced inflammation [170]. MSCs-exos transfer specific miRNAs (e.g., miR-21, miR-24, miR-146a) and proteins that can regulate cell proliferation, apoptosis, fibrosis, angiogenesis, and immune cell phenotype, thereby fostering tissue regeneration and immune suppression [160,171].

MSC-derived exosomes regulate NP cell phenotypes primarily through the delivery of specific miRNAs that modulate key signalling pathways involved in cell survival, apoptosis, and extracellular matrix homeostasis. For instance, MSC-exosomes enriched with miR-21 be taken up by NP cells, where miR-21 targets the phosphatase and tensin homolog (PTEN), thereby activating the PI3K/Akt signalling pathway to inhibit apoptosis and protect NP cells from degeneration induced by inflammatory stimuli such as TNF-α [158]. Similarly, MSC-exosomal miR-125b-5p suppresses NP cell apoptosis by targeting TRAF6 and inhibiting NF-κB activation, which attenuates intervertebral disc degeneration (IVDD) progression [172]. These miRNAs act as critical regulators by transferring from MSC-exosomes to NP cells, altering gene expression networks that promote cell proliferation, reduce fibrosis, and enhance extracellular matrix synthesis, thus maintaining NP cell phenotype and function [172,173]. This mechanism highlights the therapeutic potential of MSC-exosomes as a cell-free strategy to modulate NP cell phenotypes and treat degenerative disc diseases through miRNA-mediated molecular pathways.

Due to their lipid bilayer enriched with integrins and ligands, MSCs-exos fuse with or are internalized by recipient cells via receptor-mediated endocytosis or membrane fusion, delivering their cargo directly into the cytosol to modulate cell behaviour [174]. Lipids like prostaglandins, leukotrienes, and phosphatidylserine, along with enzymes regulating lipid metabolism, contribute to modulating homeostasis and inflammatory responses in target tissues [175]. Overall, MSCs-exosomes act as messengers that transfer functional molecules, impacting cellular signalling pathways involved in immune regulation, tissue repair, and regeneration.

### 9.3 Comparison of MSC-derived exosomes and mesenchymal stem cell therapies

MSCs are multipotent stromal cells capable of differentiating into a variety of cell types, including osteoblasts, chondrocytes, and adipocytes, making them attractive candidates for regenerative therapies such as in IVD degeneration [176]. Beyond differentiation, MSCs exert therapeutic effects primarily through their paracrine activity, secreting bioactive factors that modulate inflammation, stimulate endogenous repair mechanisms, and inhibit apoptosis [177]. Recent research has demonstrated that exosomes derived from MSCs can replicate much of the regenerative, anti-inflammatory, and immunomodulatory effects of MSCs without the risks associated with live cell transplantation [178]. Exosomes are small extracellular vesicles (30–150 nm) enriched with proteins, lipids, mRNAs, and microRNAs. They serve as natural carriers for cell-to-cell communication, mediating many of MSCs' therapeutic effects [179].

Compared to MSCs, exosomes have several key advantages. They exhibit lower immunogenicity, reducing the risk of immune rejection and eliminate the risk of tumorigenicity, which can occur from improperly differentiated or transformed MSCs [180] They exosomes are easier to store, sterilise, and standardise for clinical applications compared to living MSCs that require strict handling [179]. Exosomes avoid vascular occlusion risks associated with intravenous MSCs administration, since cells may be trapped in the lungs or other organs [178]. However, challenges remain in large-scale exosome production, standardized isolation techniques, and precise dosing strategies before widespread clinical use can be achieved [181]. Thus, while MSCs remain a powerful therapeutic tool, MSCs-derived exosomes are increasingly viewed as a safer, cell-free alternative with the potential to revolutionize regenerative medicine.

When comparing MSCs and MSC-derived exosomes (MSC-Exos) in regenerative therapy, two key factors include production costs and clinical translation stages which play a major role in shaping their therapeutic development and use. For production costs, MSC production involves cell isolation, expansion, and quality control, which are labor-intensive and costly, especially when scaling for clinical use. Traditional 2D monolayer cultures have higher costs per unit of therapeutic effect compared to advanced 3D dynamic culture systems using bioreactors and microcarriers, which enhance cell yield and reduce labor and media consumption, thus lowering costs by up to 40 % per anti-inflammatory unit [182]. In contrast, MSC-Exos are smaller, less complex, and can be produced in large quantities from MSC-conditioned media, making their production simpler and potentially less expensive. Exosomes can be isolated by ultracentrifugation or other scalable methods, and their stability allows long-term storage without activity loss, further reducing logistical costs [183]. However, large-scale, standardized exosome production remains challenging due to variability in isolation methods, yield, and purity, which complicates cost-effective manufacturing and regulatory approval [184].

For clinical translation stages, MSC therapies have advanced further in clinical translation, with numerous ongoing and completed trials demonstrating safety and efficacy in various indications. MSCs’ ability to engraft, differentiate, and modulate immune responses has been well characterized, though concerns about immune rejection and tumorigenicity persist. MSC-Exos, as cell-free therapies, offer advantages such as lower immunogenicity and no risk of tumor formation, potentially improving safety profiles [183]. Nevertheless, MSC-Exos are at earlier clinical stages, with fewer completed trials and regulatory pathways still evolving. The lack of standardized production and characterization protocols for exosomes slows their clinical adoption despite promising preclinical data [184].

Challenges in standardizing exosome production is a critical hurdle for MSC-Exos therapy. Variability arises from differences in MSC source, culture conditions, exosome isolation techniques, and characterization assays. The heterogeneity in exosome populations and cargo complicates reproducibility and potency assessment. Efforts to develop scalable bioreactor systems and robust quality control measures are underway, but consensus on best practices is lacking. Additionally, regulatory frameworks for exosome-based products are still being defined, requiring clear standards for purity, potency, and safety to facilitate clinical translation [184,185].

## 10 Gene therapy

Gene therapy entails altering gene expression inside intervertebral discs. The genes are delivered into cells via a vector, which can be injected directly into the cell or transmitted by a viral vector (Table 4). Retroviral vectors, adenoviruses, adeno-associated viruses, and baculoviruses are all widely used viral vectors [72]. Nonviral vectors under development have not yet matched the effectiveness of viral vectors. The main disadvantage of utilising a retroviral vector is the risk of insertional mutagenesis, which might result in the development of malignancies. An adenovirus vector is highly immunogenic, which might trigger a significant immune response against the foreign transgene-encoded proteins, potentially reducing the effectiveness of this approach. Preparing viral vectors for gene transposition is costly and poses potential risks to patients.

Advancements in nonviral transmission agents might reduce expenses and enhance the safety of this strategy for treating IVDD to a great extent. Microbubbles produced using sonoporation are being developed as a nonviral transmission agent. This method utilises microbubbles to transport plasmid DNA containing the desired proteins into cells by sonoporation, which creates temporary openings on the cell membrane using ultrasound. An alternative approach for intradiscal gene therapy involves reducing the activation of genes that are detrimental to the disc's equilibrium rather than increasing the anabolic cascade, which requires substantial energy [72].

Promising targets for gene therapy have included LMP-1 (regulation of BMP-7), disintegrin, MMPs, TIMPs, and chondrocyte-specific transcription factors (Ad-Sox9) [102,[156], [153], [154], [155], [157]]. In rat models, plasmid DNA was coupled with microbubbles to deliver transfected genes that remained active in cultured intervertebral discs for up to 24 weeks. In a rabbit model, increased levels of LMP-1 led to increased expression of PG, BMP-2, and BMP-7. In a separate rabbit model, larger levels of TIMPs were associated with slower degeneration. This was accompanied by increased Ad-Sox9 expression, which preserved chondrocytic features while restore matrix [188].

Research findings underscore the efficacy of gene therapy in modifying the expression of critical mediators involved in IVDD, including tumour necrosis factor-alpha (TNF-α) and interleukin-1 beta (IL-1β). Results demonstrate significant changes in marker expression, indicating a promising avenue for targeted therapeutic strategies in the management of disc degeneration. In conclusion by suggesting that gene therapy not only holds the potential for long-term therapeutic effects but also represents a tailored approach necessary for effectively addressing the molecular complexities of degenerative disc disease.

Overall, gene therapy update serves as a valuable resource for researchers and clinicians interested in the evolving landscape of gene therapy in spinal health, underscoring its promise to transform treatment paradigms for intervertebral disc degeneration [189].

### 10.1 Recent advances in non-viral delivery systems

#### 10.1.1 Nanoparticle carriers

Nanoparticle-based systems have been extensively developed to address limitations of viral vectors, including immunogenicity and packaging constraints. These systems include lipid-based, polymer-based, inorganic nanoparticles, and extracellular vesicle-based carriers. Key design considerations for nanoparticles include stability in physiological conditions, targeting efficiency to specific tissues or cells, biocompatibility, biodegradability, and efficient encapsulation of CRISPR components with mechanisms to facilitate endosomal escape [190]. For example, lipid nanoparticles composed of ionizable lipids, cholesterol, and PEGylated lipids have achieved editing efficiencies over 80 % in liver cells in vivo. Polymer-based nanoparticles, such as PEG-b-PLGA and PEI-coated DNA nanoclews, have shown transfection efficiencies up to 80 % *in vitro*. Inorganic nanoparticles like CRISPR-Gold have demonstrated up to 61.5 % encapsulation efficiency and effective gene editing in vivo. Extracellular vesicle-based nanoparticles also show promise, with up to 58 % gene knockdown *in vitro* and in vivo [190].

#### 10.1.2 Electroporation

Electroporation uses short electric pulses to transiently permeabilize cell membranes, allowing direct gene transfer. Recent micro/nano-electroporation technologies integrate micro/nanostructures to improve cell viability, transfection efficiency, and dose control. These advances enhance practicality for both *in vitro* and in vivo applications, offering a physical method that avoids risks associated with viral vectors such as insertional mutagenesis and immunogenicity. Electroporation is recognized for its high-throughput capability and ability to deliver diverse cargoes efficiently, making it an effective tool in gene therapy [191].

#### 10.1.3 CRISPR editing in regulating inflammatory pathways

CRISPR/Cas9 technology has been applied to modulate inflammatory pathways by targeting key genes involved in inflammation. Non-viral delivery systems have enabled efficient CRISPR-mediated editing to downregulate pro-inflammatory proteins. For instance, lipid-based nanoparticles delivering CRISPR/Cas9 targeting Pcsk9 achieved over 80 % editing efficiency in liver cells, which is relevant since Pcsk9 influences inflammatory responses and cardiovascular disease [190,192]. Moreover, extracellular vesicle-based delivery of CRISPR components has been used to suppress inflammatory mediators *in vitro* and in vivo, demonstrating the potential for precise regulation of inflammation through gene editing.

In summary, recent advances in non-viral delivery systems such as nanoparticle carriers and electroporation provide safer and increasingly efficient alternatives to viral vectors for CRISPR/Cas9 delivery. These systems enable targeted gene editing to regulate inflammatory pathways, opening avenues for novel therapies in inflammatory and cardiovascular diseases, with ongoing research focused on optimizing delivery efficiency and safety profiles.

Despite the higher transfection efficiency of viral vectors, concerns about immunogenicity, insertional mutagenesis, and production complexity remain significant. Non-viral vectors offer improved safety and flexibility as shown in Table 6, with ongoing improvements in delivery efficiency through nanoparticle engineering and physical methods like electroporation [[161], [162], [163]].

**Table 6:** Safety and transfection efficiency: viral vs. non-viral vectors.

Aspect	Viral Vectors	Non-Viral Vectors
**Transfection Efficiency**	Generally higher efficiency, especially in difficult-to-transfect cells; stable gene expression is possible	Lower efficiency: challenges include protection from degradation and endosomal escape
**Safety Profile**	Higher immunogenicity; risk of insertional mutagenesis leading to oncogenesis; complex production	Lower cytotoxicity and immunogenicity; minimal risk of insertional mutagenesis; easier and scalable manufacturing
**Cargo Capacity**	Limited by viral capsid size, e.g., AAV vectors have packaging constraints	Larger cargo capacity; no strict size limits
**Targeting Specificity**	Often tissue-specific tropism but can be limited; off-target risks exist	Targeting can be enhanced by nanoparticle surface modification but generally lower specificity
**Re-administration**	Limited due to immune response against viral proteins	Possible due to low immunogenicity
**Production Complexity**	Complex, costly, and requires stringent safety measures to prevent replication-competent viruses	Simpler, cost-effective, and scalable production

## 11 Clinical trials of cell-based therapy for IVD regeneration

While no definite biological foundation has been established, direct tissue substitution is becoming less desirable. The biochemical interaction between transplanted cells and recipient cells is critical, and knowing the pathways involved is a tough and critical step. Progress in assessing cell-based treatments is dependent on a thorough understanding of both the sickness and the therapeutic process. Given the growing emphasis on cell-based clinical trials to treat intervertebral disc degeneration, it is critical to assess both degenerative and regenerative elements of biology.

As of January 2024, there were 12 completed and published clinical trials, involving the strategy to use MSCs, chondrocyte and NP cells for treating lumbar IVD degeneration (Table 7). A total of 14 ongoing clinical trials provides important insights into the advancement of cell therapy testing for intervertebral disc (IVD) degeneration to date (Table 8). Among these, nine clinical trials focus on BM-MSCs, three studies on ADSCs, and two studies on umbilical cord-derived MSCs for the treatment of lumbar IVD degeneration.

**Table 7:** Completed and published clinical trials investigating cell-based therapies for IVD degeneration.

Study type	Indication	Cell type (source)	Control conditions	No. of patients	Duration (months)	Outcomes	Refs
**Stem cells**							

Phase I/II, randomized double-blind study	Lumbar disc degeneration and chronic low back pain	BM-MSCs (allogeneic)	Sham injection of anaesthetic to paravertebral musculature	24	12	No adverse events, reduction in pain and disability and improvements in Pfirrmann grade	[105]
Pilot study	Low back pain with posterior disc bulge	BM-MSCs (autologous)	None	33	60	No serious adverse events; reduction in pain scores relative to baseline at 36–72 months; reduction in posterior bulge size	[196]
Phase I/II, prospective, non-randomized open-label, single-arm study	Lumbar disc degeneration and chronic low back pain	BM-MSCs (autologous)	None	11	12	No adverse effects; reduction in pain and disability	[197]
Prospective, non-randomized, open-label, two-arm study	Discogenic low back pain	Bone marrow concentrate (autologous)	None	26	24	Reduced low back pain. sporadic increases in disc hydration	[198]
Phase I, open-label, single-arm study	Discogenic low back pain	ADSCs (autologous)	None	10	12	No adverse events, reduced pain and reduced disability; sporadic increases in hydration	[199]
Non-randomized, open label, multicentre study	Lumbar disc degeneration and chronic low back pain	Stromal vascular fraction (autologous)	None	15	12	No adverse events, improved flexion reduced pain and reduced disability	[200]
Preliminary	Chronic discogenic low back pain	UC-MSCs (allogeneic)	None	2	24	Reduced pain and disability and increased function	[201]

**Chondrocytes, NP and IVD Progenitor Cells**							

Prospective, randomized, open label, multicentre study	Single-level lumbar disc herniation	Disc-derived chondrocytes (autologous)	Discectomy alone	112	48	Reduced low back pain, decreased disability index, improved hydration in treated and adjacent IVDs and no change in disc height	[202,203]
Phase I, prospective, single-arm study	Single-level disc degeneration with low back pain	Juvenile articular chondrocytes (allogeneic)	None	15	12	Reduced low back pain and improvement on MRI	[204]
Phase I, prospective study	Disc degeneration adjacent to fused disc	Reactivated nucleus pulposus cells (autologous)	None	10	36	No adverse events and no progression of disc degeneration	[205]
Phase I, prospective, randomized, multicentre study	Single-level symptomatic lumbar disc herniation	Disc-derived chondrocytes (autologous)	Cell-free hydrogel carrier	15	1.5	No harmful material extrusion or immunological rejections	[206,207]
Prospective, randomized, parallel arm, multicentre study	Lumbar disc degeneration and chronic low back pain	Viable cellular allograft–nucleus pulposus matrix (allogeneic)	Saline injection or conservative care	24	12	Reduced low back pain and improved function	[207]

**Table 8:** Ongoing clinical trials were investigating cell-based therapies for IVD degeneration.

Trial ID	Cell type	Study design	Indication	Control conditions	No. of patients	Status	Refs
NCT01860417	BM-MSCs (allogeneic)	Phase I/II, prospective, randomized, blinded, controlled. study of 12 months	Lumbar disc degeneration and chronic low back pain	Sham injection (2 ml of 1 % mepivacaine into paravertebral musculature)	25	Completed; no results posted	[208]
EudraCT 2012-003160-44	BM-MSCs (autologous)	Phase II randomized, controlled study of 24 months	Lumbar disc degeneration and chronic low back pain	Unspecified	34	Ongoing	[209]
NCT03692221	BM-MSCs (autologous)	Early phase I, randomized, open-label study of 12 months	Symptomatic lumbar disc degeneration	Untreated healthy control	24	Not yet recruiting	[210]
NCT03737461/EudraCT 2017-002092-25	BM-MSCs (allogeneic)	Phase II/III, prospective, randomized, double-blind, multicentre study of 24 months	Lumbar disc degeneration and chronic low back pain	Sham injection (2 ml of 1 % xylocaine into paravertebral musculature)	112	Recruiting	[211]
NCT03340818	Bone marrow concentrate (autologous)	Randomized, double-blind, placebo-controlled study of 12 months	Chronic low back pain with abnormal disc pathology	Saline injection	60	Recruiting	[212]
NCT04559295	Bone marrow concentrate (autologous)	Prospective study of 24 months	Disc degeneration and low back pain	No treatment	80	Active, not recruiting	[213]
NCT03912454	Bone marrow aspirate concentrate (autologous)	Single-arm, prospective case series of 12 months	Lumbar disc degeneration and chronic low back pain	None	20	Enrolling by invitation	[214]
NCT01643681	ADSCs (autologous)	Open-label, single-arm study of 6 months	Lumbar disc degeneration and chronic low back pain	None	0	Withdrawn	[215]
NCT02338271	ADSCs (autologous)	Open-label, single-arm study of 12 months	Lumbar disc degeneration and chronic low back pain	None	10	Unknown	[216]
NCT03461458	ADSCs (autologous)	Phase I, prospective, non-randomized, dose-escalation study of 24 months	Lumbar disc degeneration and chronic low back pain	None	12	Active, not recruiting	[217,218]
NCT4414592	UC-MSCs (allogeneic)	Open-label, single-arm study of 12 months	Lumbar disc degeneration and low back pain	None	20	Recruiting	[218]
NCT04499105	UC-MSCs (allogeneic)	Open-label, single-arm study of 6 months	Degenerative disc disease with no improvement from conventional treatment	None	10	Recruiting	[219]
NCT01290367	Mesenchymal precursor cells (allogeneic)	Phase II, prospective, randomized, double-blind, controlled, multicentre study of 36 months	Lumbar disc degeneration and chronic low back pain	Sham saline injection or placebo hyaluronic acid injection	100	Completed. no results posted	[220,221]
NCT02412735	Mesenchymal precursor cells (allogeneic)	Phase III, prospective, randomized, double-blind, placebo-controlled, multicentre study of 24 months	Lumbar disc degeneration and chronic low back pain	Sham saline injection	404	Active, not recruiting	[221]

## 12 Obstacles of stem cell transplantation

Despite its immense therapeutic and preclinical usefulness, MSCs treatment has several limitations. First, several studies concluded that transplanted cells could not survive in the disc's hypoxic environment. Similarly, inflammatory mediators, the degenerated disc's low pH, low glucose levels, and hyperosmolarity may all interfere with the activation of transplanted MSCs [222]. These therapies may cause granulocytosis, graft rejection, ectopic bone growth, and microvascular embolism. After a three-month pilot study with autologous bone marrow stem cell injection, researchers discovered a substantial reduction in pain and impairment [223]. In a second investigation, autologous BM-MSCs were transplanted into the intervertebral discs of five patients with degenerative disc degeneration [149]. The data showed clinical improvement after 4–6 years of follow-up. In the Noriega et al. trial, the effect of the injected cells was practically complete at three months and was constant at six and twelve months in a group of twenty-four IVDD patients who received allogeneic BM-MSCs [224]. In addition, the MRI results showed that the disc had partially healed. In one study, researchers found that injecting allogenic hypoxic-cultured MSCs was a more effective way to maintain disc height [225].

It is crucial defining and implementing a cohort-based approach to identify donor cells, tests, culture optimization and scheduling for future research. Characterising the ideal pH condition for cell transplantation, the adequate carrier, the in vivo behaviour of MSCs, and factors that can lead to the differentiation of MSCs to annular or NP-like cells require further research [162,226]. Permission will eventually have to be gauged through human clinical trials for the safety, feasibility, and efficacy of MSCs transplantation. This new understanding is leading to new therapy options, including the development of cell-free methodologies. The therapeutic benefits of stem cells, including MSCs, can also be recapitulated by their exosomes. The inductive signals they release include a variety of paracrine mediators that are currently under investigation as a cell-free therapy for IVDD [227]. Exosomes from MSCs include several regulatory components that can prevent intervertebral disc degeneration by inhibiting apoptosis, ECM breakdown, inflammation, and promoting chondrogenic differentiation [228].

To offer successful therapy, issues such as delivery routes, cell counts, volumes, and dosing duration must be addressed. It is unknown if WJ-MSCs for cell-based therapy must be differentiated *in vitro* into the target tissue before transplantation or if they may be implanted directly into the patient and differentiated and engrafted in vivo. It is unknown if functional improvements will arise from the engraftment of differentiated tissues or from paracrine activities, comparable to autologous bone marrow MSCs transplant. Another key challenge is determining in clinical studies the efficacy of allogeneic WJ-MSCs engraftment in humans, given their shown hypoimmunogenic properties.

### 12.1 Ethical Controversies surrounding iPSCs and cloning technologies

Induced pluripotent stem cells (iPSCs) have revolutionized regenerative medicine by providing an ethically favourable alternative to embryonic stem cells as they do not require the destruction of embryos [229]. However, iPSCs introduce new ethical challenges, particularly concerning the potential misuse of cloning technologies. The ability of iPSCs to generate patient-specific pluripotent cells raises concerns about human reproductive cloning and the creation of genetically modified embryos or human-animal chimeras [230]. These possibilities provoke profound ethical debates centred on human identity, dignity, and the moral limits of genetic manipulation. Furthermore, the reprogramming process involves genetic and epigenetic modifications, which may have unpredictable consequences, emphasizing the need for ongoing ethical oversight [231].

### 12.2 Immunogenicity of allogeneic mesenchymal stem cells (MSCs)

Mesenchymal stem cells (MSCs) are widely used in regenerative therapies due to their immunomodulatory properties and relatively low immunogenicity. Nonetheless, emerging evidence indicates that allogeneic MSCs are not completely immune-privileged. Studies have demonstrated that allogeneic MSCs can elicit immune responses, including activation of T cells and natural killer cells, which may lead to rejection or reduced therapeutic efficacy [232]. Additionally, some reports suggest that MSCs might contribute to tumor progression or metastasis in certain contexts, raising safety concerns for their clinical use [233]. These findings highlight the importance of careful donor selection, immunological matching, and long-term monitoring in MSC-based therapies.

### 12.3 Tumorigenicity and immunogenicity of iPSCs

While iPSCs offer the advantage of patient-specific therapy, their tumorigenic potential remains a significant obstacle. The reprogramming process can introduce genetic mutations or epigenetic abnormalities, increasing the risk of teratoma formation and malignant transformation after transplantation [234]. Moreover, despite being autologous, iPSC derivatives may express aberrant antigens or incomplete differentiation markers that trigger immune rejection [235]. These dual risks of tumorigenicity and immunogenicity necessitate comprehensive characterization and purification of iPSC-derived cell populations before clinical application to ensure safety.

### 12.4 Standardization of preclinical safety evaluations

Given the ethical and biological risks associated with iPSC and MSC therapies, establishing rigorous and standardized preclinical safety evaluation protocols is imperative. Such standards should include assessments of genetic stability, differentiation fidelity, tumorigenic potential, and immunogenicity using validated *in vitro* and in vivo models [236]. Regulatory frameworks must mandate these evaluations to minimize adverse outcomes and facilitate safe clinical translation. Furthermore, transparency in reporting and long-term follow-up studies are essential to monitor potential late-onset effects and improve therapeutic protocols.

## 13 Conclusions

Intervertebral disc degeneration remains a major clinical challenge due to its multifactorial aetiology and limited regenerative capacity. A comprehensive investigation and knowledge of disorders are critical for finding and creating appropriate treatment strategies. Additional research is needed to explain the root causes of degeneration and develop new treatment strategies based on current knowledge. An interdisciplinary strategy that encompasses all elements of spinal research, from fundamental science to practical therapeutic applications, is required. These multimodal regenerative strategies will lead to novel IVD degeneration therapies. Current research has made significant strides in the development of biomaterials and cell-based therapies, offering promising avenues for restoring disc structure and function. Looking forward, the integration of gene therapy holds potential for precise modulation of molecular pathways involved in IVD degeneration. By precisely editing genes underpinning molecular mechanisms of degeneration, CRISPR could enhance the regenerative capacity of the IVD, ultimately alleviate pain. Likewise, the cutting-edge of 3D bioprinting in IVD tissue engineering enables the fabrication of anatomically accurate, patient-specific IVD constructs that closely mimic native tissue architecture. Innovative biomimetic scaffolds with enhanced bioactivity and mechanical properties continue to evolve, improving cell viability and integration. Furthermore, AI-driven diagnostics are emerging as powerful tools for early detection, personalized treatment planning, and prediction of therapeutic outcomes. Future research should focus on the convergence of these advanced strategies to develop comprehensive, minimally invasive, and durable solutions for IVD degeneration, ultimately improving patient care and quality of life.

## Declaration of competing interest

The author(s) declare no conflict of interest.
